# High-throughput screening of cell-free riboswitches by fluorescence-activated droplet sorting

**DOI:** 10.1093/nar/gkac152

**Published:** 2022-03-07

**Authors:** Takeshi Tabuchi, Yohei Yokobayashi

**Affiliations:** Nucleic Acid Chemistry and Engineering Unit, Okinawa Institute of Science and Technology Graduate University, Onna, Okinawa 904-0495, Japan; Nucleic Acid Chemistry and Engineering Unit, Okinawa Institute of Science and Technology Graduate University, Onna, Okinawa 904-0495, Japan

## Abstract

Cell-free systems that display complex functions without using living cells are emerging as new platforms to test our understanding of biological systems as well as for practical applications such as biosensors and biomanufacturing. Those that use cell-free protein synthesis (CFPS) systems to enable genetically programmed protein synthesis have relied on genetic regulatory components found or engineered in living cells. However, biological constraints such as cell permeability, metabolic stability, and toxicity of signaling molecules prevent development of cell-free devices using living cells even if cell-free systems are not subject to such constraints. Efforts to engineer regulatory components directly in CFPS systems thus far have been based on low-throughput experimental approaches, limiting the availability of basic components to build cell-free systems with diverse functions. Here, we report a high-throughput screening method to engineer cell-free riboswitches that respond to small molecules. Droplet-sorting of riboswitch variants in a CFPS system rapidly identified cell-free riboswitches that respond to compounds that are not amenable to bacterial screening methods. Finally, we used a histamine riboswitch to demonstrate chemical communication between cell-sized droplets.

## Graphical Abstract

**Graphical Abstract:**
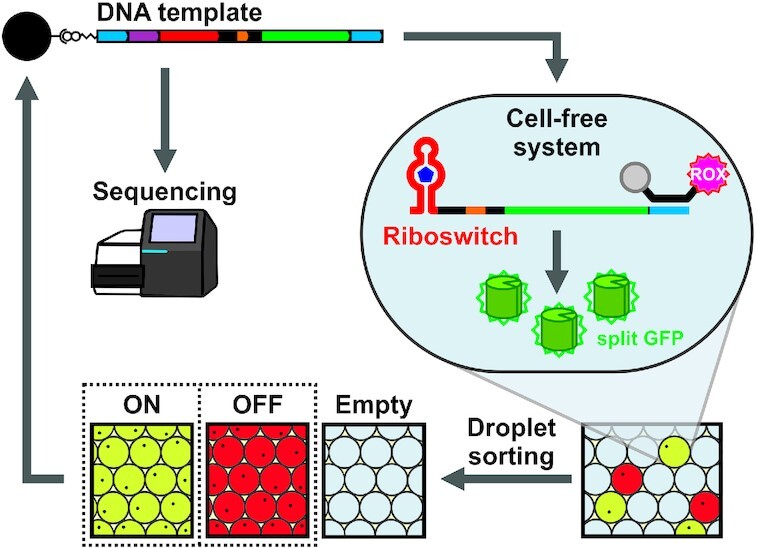
Cell-free riboswitches that function in a cell-free protein synthesis system were engineered by fluorescence-activated droplet sorting. The riboswitches were used to enable chemical communication between cell-sized droplets.

## INTRODUCTION

In the emerging field of cell-free synthetic biology, researchers aim to build complex biomolecular systems that mimic or are inspired by biological systems without using living cells (1–3). Bottom-up construction of biological systems tests our understanding of their design principles. Moreover, cell-free systems are subject to fewer experimental constraints compared to living cells, thus allowing more control over the experimental parameters. Therefore, functions that are difficult or impossible to implement in living cells may be possible with cell-free systems, for example, production of toxic compounds or artificial cells with compartments made of synthetic polymers.

Cell-free protein synthesis (CFPS) systems based on cell lysates (i.e. S30 extract) (4–7) or reconstituted biomolecular components (i.e. PURE system) (8,9) are used to build genetically programmed cell-free systems. As is the case with living cells, cell-free systems based on CFPS systems require genetic parts and devices such as chemically responsive gene switches to implement complex functions. However, such genetic components are almost always borrowed from those of living cells or engineered and optimized in living cells. Consequently, they may not function optimally in a cell-free environment, and their performance and availability are subject to the biological constraints of living cells.

Current options for chemical regulation in CFPS systems are limited. Many systems have used canonical transcription factor-based systems, such as LacI/isopropyl β-D-thiogalactopyranoside (IPTG), as a generic inducible platform to activate protein synthesis in CFPS systems (10–13). However, there are a number of limitations in using transcription factor-based gene switches in cell-free systems. For example, the switch performance is dependent on the levels of the transcription factor and the DNA template. Expression of the transcription factor *in situ* can affect the response time, and it may also impose a significant metabolic burden on the translational machinery (14,15). Furthermore, despite recent advances, engineering transcription factors to respond to new trigger molecules remains challenging (16–19).

As an alternative strategy to enable cell-free systems to respond to chemical signals, riboswitches have attracted increasing attention. Mainly found in bacteria, riboswitches are regulatory RNA sequences located in the untranslated regions (UTRs) of mRNAs that can upregulate or downregulate gene expression in response to specific chemical signals without protein factors (20–25). A canonical riboswitch contains an aptamer domain that specifically recognizes a ligand molecule, and an expression platform that mediates a structural change upon aptamer–ligand interaction resulting in modulation of protein expression (26). Because it is known that RNA aptamers that recognize diverse molecules can be obtained by *in vitro* selection (SELEX), researchers have developed synthetic riboswitches that respond to natural and non-natural chemical signals using aptamers selected in the laboratory. Numerous synthetic riboswitches have been engineered that function in bacteria and mammalian cells, as well as in other cells and organisms (27–31). In reality, however, the variety of aptamer–ligand combinations employed as synthetic riboswitches is limited due to biological constraints such as cell permeability, toxicity, and compromised aptamer–ligand binding in the complex cellular milieu.

Riboswitches have several potential advantages as chemical interfaces for cell-free systems which make them attractive tools for synthetic biology and other applications (27,30,32,33). Riboswitches are *cis*-regulatory elements embedded in the same mRNA of the gene whose expression is regulated; therefore, riboswitches subject to fewer off-target effects and respond faster compared to transcription factor-based switches. Without protein components, riboswitches impose a minimal burden on the translational machinery. Additionally, the cell-free platforms may circumvent some of the biological constraints that have limited many *in vitro* selected aptamer–ligand combinations to be used as riboswitches in living cells, such as ligands exhibiting low cell permeability, low stability inside cells (rapidly metabolized), or high toxicity.

A number of cell-free riboswitches have been reported in the literature, most of them for the purpose of understanding or demonstrating how aptamer–small molecule interactions affect gene expression (34). More recently, cell-free riboswitches have been used as components in cell-free systems with sophisticated or practical functions. For example, some groups used a theophylline riboswitch that was designed for *Escherichia coli* to implement chemical communication between artificial cells (35–37). We recently developed a novel aptamer that binds histamine and used it to engineer cell-free riboswitches that function in PURE system and artificial cells (38). Thavarajah et al. adapted a natural riboswitch to fabricate a portable cell-free sensor for field measurement of fluoride in drinking water sources (39).

Despite these examples, design of cell-free riboswitches is not straightforward. Most of the cell-free riboswitches reported to date were: 1) based on natural riboswitches, 2) originally developed in *E. coli* using high-throughput screening or selection, or 3) designed by trial-and-error. A notable exception is the computational design strategy by the Salis group (40) who designed multiple cell-free riboswitches using several aptamers. Although several strategies for high-throughput screening and selection of riboswitches *in vivo* have been successfully used to engineer bacterial riboswitches (41–44), they often do not function as well in cell-free systems (35–37). Furthermore, as noted above, many aptamer–ligand combinations are not amenable to bacterial screening or selection. The lack of high-throughput screening strategies for optimizing cell-free riboswitches directly in CFPS systems strongly limits the variety and the performance of cell-free riboswitches that can extend the capabilities of cell-free systems.

Here, we report the first high-throughput screening strategy for cell-free riboswitches based on water-in-oil droplet sorting in microfluidic chips. We used the method to develop three types of riboswitches based on two aptamers. Finally, we show that the riboswitches optimized under cell-free conditions can be used to implement chemical communication between sender and receiver droplets.

## MATERIALS AND METHODS

### DNA amplification

PCR was performed using Q5 High-Fidelity 2X Master Mix (New England Biolabs) with 0.5 μM of each primer unless noted otherwise. PCR products were purified by silica columns (DNA Clean & Concentrator Kit-5, Zymo Research) or, when necessary, by agarose gel electrophoresis (Zymoclean Gel DNA Recovery Kit, Zymo Research).

### Template construction

**Figure 1.**
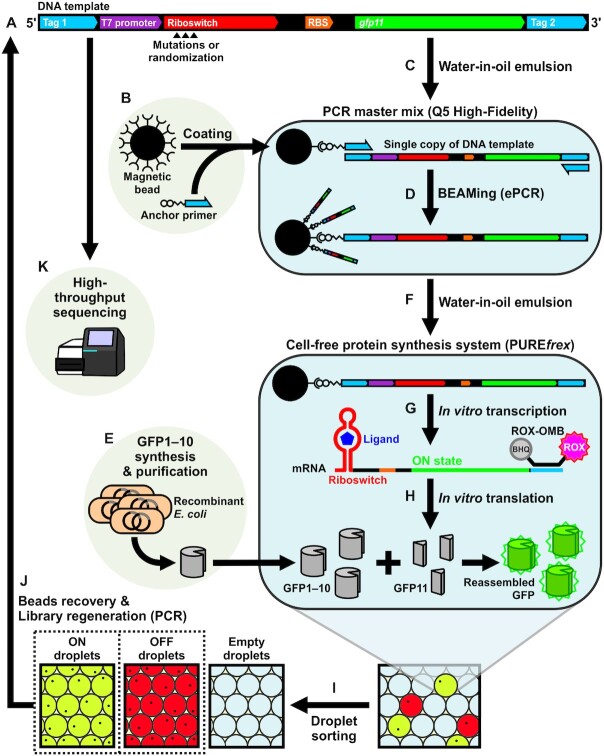
Overview of the high-throughput screening strategy for cell-free riboswitches. (**A**) DNA template encoding the riboswitch variants. (**B**) Streptavidin-coated magnetic microbeads are used to immobilize a biotinylated primer. (**C, D**) A single DNA template and a magnetic bead are encapsulated in a water-in-oil emulsion for clonal amplification of the DNA template on the magnetic bead (BEAMing). ePCR: emulsion PCR. (**E**) Recombinant GFP1–10 fragment is produced in *E. coli*. (**F, G, H**) The DNA template-immobilized magnetic beads are re-encapsulated in water-in-oil emulsions containing the CFPS system and GFP1–10 fragment. Droplets that produce mRNAs yield ROX fluorescence. Droplets that contain riboswitch variants at ON state yield green fluorescence due to assembly of the split GFP fragments. ROX-OMB: ROX-labeled molecular beacon. (**I**) The droplets with ROX fluorescence are sorted for ON or OFF states of the riboswitch variants. (**J**) The magnetic beads are recovered from the sorted droplets and amplified by PCR for the next round of sorting. (**K**) The enriched riboswitch candidate sequences are analyzed by high-throughput sequencing.

Each riboswitch construct consists of a 5′sequence to which the anchor primer P0L and forward primer P3L anneal (tag 1), T7 promoter, riboswitch sequence, optimized ribosome binding site (RBS), *gfp11* gene, and a 3′ UTR sequence (tag 2) which serves as the binding site for the reverse primer P4L and the ROX-OMB molecular beacon (Figure 1A, Supplementary Table S1). The DNA templates for the GFP11 constructs were assembled by PCR from 3 or 4 overlapping oligonucleotides. The randomized libraries were constructed similarly using oligonucleotides containing 3–6 degenerate bases (N) at the desired positions. For the eGFP reporter constructs, the constant region containing the eGFP coding sequence was first cloned into pMD20 vector (TaKaRa). Each DNA template was produced by PCR using a forward primer that contains the respective riboswitch variant sequence and a common reverse primer (P-eGFP-R, Supplementary Table S1) with the eGFP-containing plasmid as a template. The PCR products were purified with silica columns or by agarose gel electrophoresis.

### Droplet generation

The water-in-oil emulsion droplets were generated by On-chip Droplet Generator (On-chip Biotechnologies) in 2D chip-800DG chips at 4°C. As the oil phase, 5% Pico-Surf 1 (Sphere Fluidics) in HFE-7500 3M Novec Engineered Fluid (Fluorochem) was used, and the aqueous phase was either a PCR mix (Q5 High-Fidelity 2X Master Mix, New England Biolabs) or a cell-free protein synthesis (CFPS) reaction mix (PURE*frex* 1.0, Gene Frontier). The sample and the oil pressures were set to 58 kPa and 79 kPa, respectively. The throughput was approximately 2.2 × 10^6^ droplets per 10 minutes, and the monodisperse droplets produced were approximately 26 μm in diameter.

### Single-template amplification on magnetic beads (BEAMing)

The template DNAs encoding the riboswitch variants were clonally amplified by emulsion PCR on magnetic beads according to the BEAMing (45) protocol with few modifications. Streptavidin-conjugated magnetic beads (Dynabeads MyOne Streptavidin C1, Invitrogen) were coated with the anchor primer P0L (Supplementary Table S1) as described previously (45). The anchor primer was modified at the 5′ end with a dual-biotin tag connected via a hexa-ethyleneglycol (18-atom) spacer (iSp18) synthesized by Integrated DNA Technologies. Water-in-oil emulsion droplets were generated as described above using an aqueous phase containing the DNA template (50 fM), P0L-coated magnetic beads (∼5 × 10^5^/μl), PCR mix, and the primers (50 nM P3L and 4 μM Texas Red-labeled P4L, Supplementary Table S1). The droplets were subjected to 35 cycles of PCR (two-step: 98°C for 10 s followed by 72°C for 10 s). To ensure that the majority of the beads are monoclonal, the DNA concentration was empirically adjusted to yield ∼10–30% positive beads (Texas Red-positive), taking Poisson distribution into account (46,47). Upon completion of the PCR, the emulsion was broken using an anti-static gun (MILTY Zerostat 3), then the magnetic beads were collected using a home-made neodymium magnet stand and washed once with the breaking buffer (15 mM Tris-HC pH 7.5, 0.5 mM EDTA, 50 mM NaCl, 25 mM KCl, 0.5% Triton-X 100, 0.5% SDS). The beads were further washed five times with TK buffer (20 mM Tris-HCl pH 8.4, 50 mM KCl) and suspended in TEK buffer (15 mM Tris-HCl pH 7.5, 25 mM KCl, 0.5 mM EDTA) for storage at 4°C.

### Cell-free protein synthesis in droplets

Water-in-oil emulsion droplets were generated as described above using an aqueous phase that contains magnetic beads coated with the DNA encoding riboswitch-*gfp11*, PURE*frex* 1.0 reaction mix, GFP1–10 (50 μg/ml, approximately 26 μM), ROX-labeled 2′-O-methyl-RNA molecular beacon (ROX-OMB) (0.5 μM), and where applicable, histamine or ciprofloxacin at an appropriate concentration. To ensure that the majority of the droplets contain only one riboswitch variant (one bead) or are empty, the concentration of the beads was empirically adjusted to yield ∼10–30% transcriptionally active droplets (ROX-positive). The emulsion was incubated at 37°C for 4 h to allow CFPS reaction to proceed.

### Droplet sorting

The droplets containing the CFPS reaction mix were sorted by fluorescence-activated droplet sorting (FADS) using an On-chip Sort instrument (On-chip Biotechnologies) in Chip-Z1001 chips. The sheath fluid used was 0.1% Pico-Surf 1 in HFE-7500, and low-density mineral oil (Sigma) was added to the collection reservoir to trap the sorted droplets and facilitate their recovery. The droplets were sorted according to ROX (FL-5, ex. 561 nm, em. 676 ± 37 nm) and GFP (FL-2, ex. 488 nm, em. 543 ± 22 nm) fluorescence. ROX fluorescence was used to detect the presence of transcriptional activity. GFP fluorescence was used to detect the riboswitch output. Examples of the dual color fluorescence density plots (FL-2 vs. FL-5) and selection gates of the droplet sorting experiments are shown in Supplementary Figures S1 and S2. The thresholds were established using the output of the empty droplets (GFP- ROX-) as the baseline. The empty droplets can be easily identified as they represent more than 70% of the droplets and always cluster at the bottom-left corner of the FL-2 vs. FL-5 plot (Supplementary Figures S1 and S2). The gates for sorting were set according to the targeted riboswitch output (ON or OFF) and adjusted for the population distribution of each library. The gates were arbitrarily set to select the ROX-positive droplets with the highest or the lowest GFP fluorescence, representing no more than 10% of the total droplet population (typically between 0.1% and 3%) (Supplementary Figure S2). The sorting gates were adjusted every cycle to increase the stringency of selection.

### Beads recovery and library regeneration

The sorted droplets were carefully recovered from the collection reservoir and transferred to a new tube. TK buffer (30 μl) and blank magnetic beads (0.5 μl) were added to the tube to improve the recovery yield. The emulsion was broken using an anti-static gun, and the magnetic beads were collected using a magnetic stand. The beads were washed once with the breaking buffer and three times with TK buffer. The washed beads were resuspended in 12 μl of the PCR reaction mix (Q5 High-Fidelity PCR Master Mix containing primers P3L and P4L). Thirty cycles of PCR (two-step: 98°C for 10 s followed by 72°C for 10 s) was performed to regenerate the riboswitch library. The PCR product was purified using a silica column or agarose gel electrophoresis. The purified DNA template was then used for the next cycle of BEAMing.

### High-throughput sequencing

To link each library with its corresponding sorting cycle, a custom DNA barcode (6–8 nt) was inserted during 6 cycles of PCR (98°C for 10 s, 69°C for 10 s, and 72°C for 15 s) using primers P-HA-Nova-T1 and P-HA-Nova-B1 (Supplementary Table S1) with the library DNA (25 nM) as template. The PCR product was column-purified and was used to produce the sequencing libraries. The final sequencing libraries were generated by PCR in 20 μl volume containing barcoded PCR product (0.5 ng) and primers TruSeq-i5 and TruSeq-i7 (Supplementary Table S1). Twelve cycles of PCR (98°C for 10 s, 69°C for 10 s, 72°C for 15 s) were performed, and the PCR product was purified by agarose gel electrophoresis. The sequencing was performed using Illumina MiSeq (150 bp, single-end) or Illumina NovaSeq (150 bp, paired-end) (Supplementary Table S3). The sequencing data was processed using a custom script. Briefly, the raw reads were merged (if paired-end reads were used) and sorted according to the corresponding droplet sorting cycles based on the custom barcode sequence introduced during the sequencing library preparation. The reads were filtered to remove those containing low quality base calls and errors in unintended positions. Finally, the number of reads for each variant was counted and expressed as a percentage of the total number of reads within each cycle (abundance). The variants were ranked by their ‘enrichment trend’ which was calculated based on the slope of the simple linear regression of the abundance of each variant through the sorting cycles.

### Mock sorting

Droplets simulating the ON and the OFF riboswitch outputs were generated using DNA templates with a ‘strong’ (P_T7_-RBS-GFP11) or a ‘weak’ RBS (P_T7_-wRBS-GFP11), respectively (Supplementary Table S2). Additionally, the DNA template for the ON-droplets contains an EcoRI restriction site while that of the OFF-droplets contains a SacI site. The DNA templates were mixed in approximately 5:95 or 95:5 ratios and were used to produce mock libraries by BEAMing and CFPS reactions as described above. The droplets were sorted, and the enrichment efficiencies were estimated by flow cytometry, fluorescence microscopy, and restriction digestion of the DNA recovered from the sorted droplets. The sorting efficiency was estimated from the droplet statistics by dividing the number of sorting events of the desired droplets by the total number of the droplets detected by the sorter within the selected sorting gate. The sorting purity, or the percentage of the droplets exhibiting the desired fluorescence output (ON or OFF) after sorting, was estimated by fluorescence microscopy.

### Restriction digestion analysis

Because the ON and OFF templates used in mock sorting tended to form a significant amount of heteroduplex products after standard PCR, the following steps were included to reduce such products before restriction digestion analysis. The templates (0.5 nM) were re-amplified with 10 cycles of PCR (98°C for 10 s, 72°C for 10 s) with primers P3L and P4L (Supplementary Table S1). The product was then diluted 10-fold with fresh PCR reaction mix of the same composition except with 2.5 × DNA polymerase and was incubated at 98°C for 2 min followed by 72°C for 2 min. The PCR products were purified with silica columns and 20 ng of each of the PCR products was digested with EcoRI-HF (New England Biolabs) and SacI-HF (New England Biolabs) according to the manufacturer's instruction. Native polyacrylamide gel electrophoresis (PAGE, 8%) was performed to separate the digested samples. The gels were stained with SYBR Gold (Invitrogen) and photographed by LuminoGraph WSE-6100Z imager (ATTO). The ratios of the ON and the OFF templates before and after sorting were estimated from the band intensities analyzed by ImageJ 1.52p.

### GFP1–10 synthesis and purification

The *gfp1–10* gene fragment was amplified from pk-thiC#19-gfp(1–10) plasmid (48) by PCR and was subcloned into pTrcHis vector. The plasmid was transformed into *Escherichia coli* BL21 (DE3). Protein expression was induced by addition of IPTG (0.5 mM) after the bacterial culture reached an OD_600_∼0.6 in LB medium supplemented with 100 μg/ml ampicillin. The cells were further incubated for 3 h at 37°C before they were harvested and lysed. The His-tagged GFP1–10 protein was purified using Ni-NTA Magnetic Agarose Beads (QIAGEN) according to the manufacturer's protocol. The protein was desalted using Amicon Ultra-0.5 ml (MWCO 3 kDa) centrifugal filters (Merck Millipore) and stored at -80°C in storage buffer (10 mM Tris-HCl pH 7.5, 1 mM EDTA, 20 mM KCl).

### Post-sequencing riboswitch evaluation

The promising riboswitch candidates from the sequencing data were individually synthetized and tested in solution. First, the riboswitch variants controlling the *gfp11* gene were evaluated in PURE*frex* 1.0 reaction mix (8 μl total reaction volume) containing the DNA template (20 nM), ROX-labeled molecular beacon (0.5 μM), GFP1–10 (50 μg/ml, approximately 26 μM), and where appropriate, the ligand (histamine or ciprofloxacin) at the indicated concentration. The samples were incubated at 37°C for 4 h in 0.2 ml PCR tubes and then transferred to a 384-well microplate (Greiner Bio-One, black, non-binding, flat-bottom). GFP fluorescence (ex. 484 nm, em. 510 nm) and ROX fluorescence (ex. 587 nm, em. 599 nm) were measured using Infinite M1000 Pro (Tecan). The GFP fluorescence values were normalized by their ROX fluorescence, and then further normalized by that of the no-riboswitch positive control (‘strong’ RBS only: P_T7_-RBS-GFP11 or P_T7_-RBS-eGFP). Some of the validated riboswitch variants were further characterized after fusing them to the *egfp* gene. CFPS reactions were performed as described above except without GFP1–10 protein. The structures shown in this article were based on published structures and computational simulations using ViennaRNA (49) web server. The free energies of the riboswitch structures were calculated using ViennaRNA/RNAfold (49–51).

### Droplet-droplet communication

The receiver droplets were produced as described above by encapsulating PURE*frex* 2.0 reaction mix (Gene Frontier) containing HA-C1g-19-GFP11 riboswitch DNA template and P_T7_-RBS-GFP(1–10) DNA template for constitutive production of GFP1–10 fragment (50 nM each). We decided to use the split GFP system instead of eGFP because we found it exhibits stronger fluorescence signal in the droplets. The sender droplets contained PURE*frex* 2.0, DNA template P_T7_-*hdc*-6xHis encoding histamine decarboxylase (*hdc*) (25 nM), ROX-labeled molecular beacon (1 μM), L-histidine (50 mM), and pyridoxal phosphate (PLP) (20 μM). Sender droplets without PLP were used as a negative control, and droplets supplemented with 50 mM histamine instead of L-histidine were used as a positive control. The *hdc* gene used for this experiment was derived from *Raoultella planticola* (ATCC 43176), but the codons were optimized for translation in *Escherichia coli* (Supplementary Table S2). The droplets were mixed in appropriate ratios and incubated at 37°C for 4 h inside the channels (250 μm × 30 μm) of 2D chip-800DG chips (On-chip Biotechnologies). The droplets were observed under an EVOS FL digital inverted fluorescence microscope (Thermo Fisher Scientific). The relative fluorescence intensity of the droplets was estimated from the raw fluorescent micrographs using ImageJ 1.52p. The intensity and the contrast of the fluorescence images presented (Figure 8B–E) were uniformly adjusted for better visualization using the microscope's built-in software and Adobe Photoshop CS6.

## RESULTS

### Overview of the cell-free riboswitch sorting strategy

Our strategy for screening a large number of cell-free riboswitch variants is illustrated in Figure 1. First, DNA templates encoding riboswitch variants are clonally amplified on magnetic microbeads following the previously reported strategy called BEAMing (Beads, Emulsion, Amplification, and Magnetics) (45) (Figure 1D). Then, the magnetic beads are individually encapsulated in water-in-oil emulsion droplets containing the CFPS system (PURE*frex*) with or without the ligand (Figure 1F). Individual droplets (containing one riboswitch variant per droplet) express a fluorescence reporter gene depending on the riboswitch output (Figure 1G, H). The droplets are sorted to recover the riboswitch-encoding templates that show the desired output (ON or OFF) (Figure 1I, J). These ON and OFF sorting cycles are repeated to enrich functional riboswitches from the original library.

Each DNA template encoding a riboswitch variant is flanked by tag 1 and tag 2 sequences that serve as primer binding sites for polymerase chain reaction (PCR) (Figure 1A). The T7 promoter is located downstream of tag 1 which drives the transcription of the mRNA by the T7 RNA polymerase. The transcript encoded downstream of the T7 promoter contains the riboswitch variant in the 5′ UTR followed by a ribosome binding site (RBS), a short open reading frame (ORF) encoding *gfp11*, and finally the tag 2 after the stop codon (Figure 1A). The droplets contain a molecular beacon that anneals to the tag 2 region (3′ UTR) of the mRNA to activate fluorescence of 6-carboxy-X-rhodamine (ROX) that serves as an indicator of active transcription. Consequently, only ROX + droplets are sorted to avoid recovering empty droplets lacking DNA template. The droplets also contain GFP1–10 fragment that noncovalently associates with the GFP11 peptide encoded in the ORF reconstituting the GFP fluorescence which reports the riboswitch output (52). This split-GFP reporter system was adopted because the efficiency of the BEAMing protocol decreases as the DNA size increases (>200 bp) (45).

### Mock sorting

**Figure 2.**
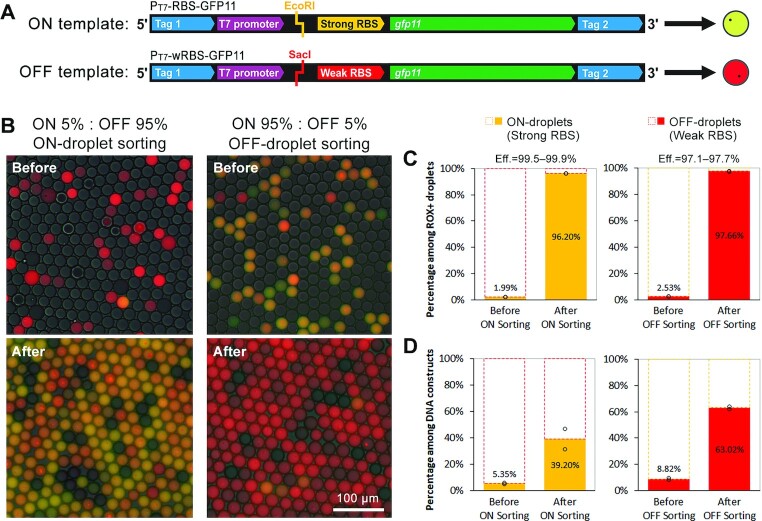
Mock sorting of ON- and OFF-droplets. (**A**) The ON template (P_T7_-RBS-GFP11) contains a ‘strong’ RBS that regulates translation of the GFP11 peptide. It also contains an EcoRI restriction site upstream of the RBS. The OFF template (P_T7_-wRBS-GFP11) contains a ‘weak’ RBS to simulate riboswitch variants in an OFF state. It also contains a restriction site recognized by SacI upstream of the RBS. (**B**) Fluorescence micrographs (merged: GFP + ROX + bright field) before and after droplet sorting for ON- (left) or OFF-droplets (right). (**C**) Abundance of ON- or OFF-droplets before and after sorting as estimated by fluorescence microscopy and flow cytometry. (**D**) Abundance of ON or OFF templates before sorting and after sorting/PCR as estimated by restriction digestion and gel electrophoresis (Supplementary Figure S1). Two independent mock sorting experiments were performed. The open circles in **C** and **D** represent the two measurements, and the bars represent the average values. Eff.: sorting efficiency or recovery rate of the desired droplets.

To validate the sorting strategy, we designed and performed mock sorting experiments using two DNA templates that simulate ON and OFF riboswitch outputs. The ON template contains a canonical RBS sequence that robustly translates the encoded gene (P_T7_-RBS-GFP11), and the OFF template contains a weakened RBS sequence with low translation efficiency (P_T7_-wRBS-GFP11) (Figure 2A, Supplementary Table S2). The ON and OFF templates were mixed in approximately 5:95 and 95:5 ratios to prepare mock libraries. The DNA mixtures were clonally amplified on magnetic beads by BEAMing, and they were used to make droplets containing single magnetic beads. The droplets were sorted for the minor member of the mock library. The sorted droplets were then imaged by fluorescence microscopy. The template DNAs were also recovered from the sorted droplets by PCR, and the relative abundance of the two species (ON and OFF) was estimated by restriction digestion of the PCR product (Supplementary Figure S3). Two independent mock sorting experiments were performed.

Fluorescence images of the droplets before and after sorting showed that ON- and OFF-droplets were enriched on average 48- and 38-fold, respectively (Figure 2C). It is worth noting that more than 70% of the initial droplets were empty to ensure that the majority of the droplets contained one magnetic bead (or none) based on the Poisson distribution. The sorting efficiently eliminated these empty droplets because we included a molecular beacon to detect droplets that exhibit active transcription. The sorting efficiency, or the recovery rate of the desired droplets in the mock libraries, was 99.5–99.9% and 97.1–97.7% for ON- and OFF-droplet sorting, respectively, as estimated from the statistical data of the measured droplets. The proportion of the desired droplets after sorting, or the sorting purity, was 79.9–82.5% (ON-droplet sorting) and 86.5–89.3% (OFF-droplet sorting) with most of the co-sorted droplets being empty droplets (Figure 2B).

Using the magnetic beads isolated from the sorted beads, we recovered template DNA by PCR. Enrichment of the ON and OFF DNA templates were estimated by restriction digestion analysis of the PCR products (Figure 2D, Supplementary Figure S3). We observed ∼7-fold enrichment for both ON and OFF templates. The lower enrichment of the DNA templates compared to the droplets is likely due to some droplets containing multiple magnetic beads as the beads tend to aggregate (Supplementary Figure S4). Therefore, some OFF templates may be co-sorted along with ON templates within the same droplet during the ON-sorting process. Conversely, some OFF templates may not be recovered due to coexisting ON templates within the same droplet during the OFF-sorting step. Nevertheless, we confirmed that DNA templates in a CFPS system can be enriched for either ON or OFF gene expression using our droplet sorting method.

### Histamine ON-riboswitches

After confirming that both ON and OFF outputs can be enriched efficiently, we set out to isolate cell-free riboswitches that respond to histamine (HA) by activating gene expression (ON-switch). Histamine is a hydrophilic vasoactive amine involved in local immune response, inflammatory processes, and allergic reactions. In the human body, it also regulates gut functions and acts as a neurotransmitter (53–55). We recently discovered an aptamer that specifically binds histamine with a dissociation constant (K_d_) of 371 nM (38). However, we were not able to execute *E. coli*-based riboswitch selection due to histamine's low bioavailability. Consequently, to engineer cell-free riboswitches that respond to histamine we initially resorted to a semi-rational design approach that involved time consuming trial-and-error (38).

**Figure 3.**
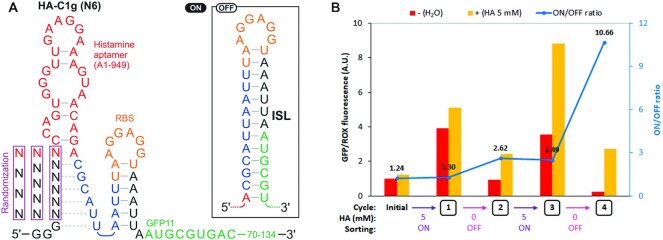
Histamine-responsive riboswitch library HA-C1g (N6). (**A**) The library design and the hypothetical structures. Formation of the inhibitory stem loop (ISL) is expected to repress translation by sequestering the RBS and the start codon. The randomized region was expected to compete with ISL by interacting with the 5′ portion of the ISL in the presence of histamine. (**B**) The enrichment of riboswitches after each sorting cycle is evidenced by the increase of the bulk ON/OFF ratio of the library. HA: histamine.

We designed a new riboswitch library based on the histamine aptamer A1-949 (38) which was fused to a linker sequence designed to sequester the region spanning the RBS and the start codon (inhibitory stem-loop, ISL in Figure 3A) resulting in repressed protein expression. Four to six nucleotides directly upstream of the aptamer were randomized with an expectation that some of the variants would activate translation when the aptamer binds histamine by competing with and disrupting the ISL structure. The library contained up to 5376 variants.

**Figure 4.**
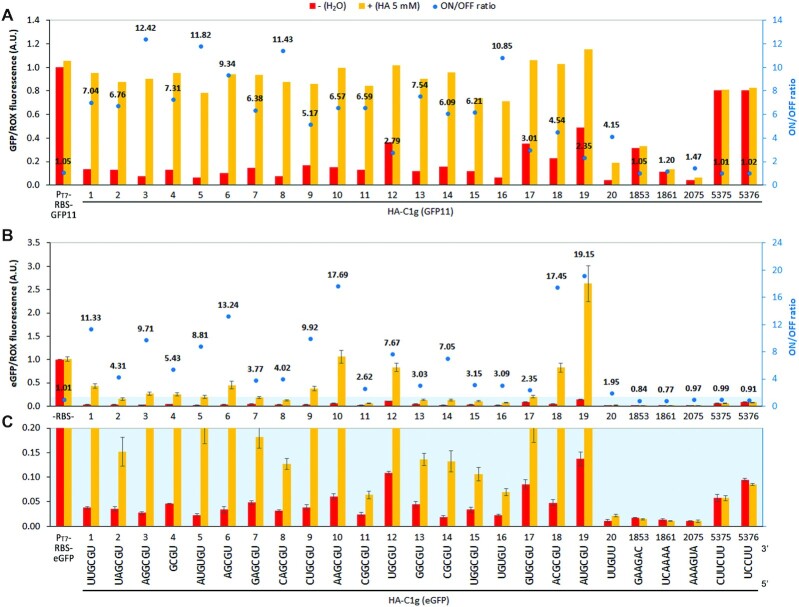
Screening of individual riboswitch variants (histamine ON-switches). (**A**) Primary screening of the riboswitch variants using GFP11/GFP1–10 (split GFP) assay. Performed once for rapid screening. The expression levels were normalized by the no-riboswitch control P_T7_-RBS-GFP11 in the absence of histamine. (**B**) Secondary screening of the riboswitch variants fused to full-length eGFP as a reporter gene. (**C**) Expanded view of the region highlighted in light blue in **B**. (**B, C**) Three independent assays were performed with the error bars representing standard deviations. The expression levels were normalized by the no-riboswitch control P_T7_-RBS-eGFP in the absence of histamine. The variant number corresponds to the respective ranking based on the enrichment trend. HA: histamine.

We performed four rounds of sorting, or two rounds each of an ON-sorting in the presence of 5 mM histamine and an OFF-sorting without histamine in alternating cycles (Figure 3B). Although the initial library showed no response to histamine when assayed as a mixture, the riboswitch library after four rounds of sorting showed 10.8 × activation in the presence of histamine, suggesting that the sequential sorting rounds enriched histamine ON-switches. The riboswitch populations from all sorting rounds were analyzed by high-throughput sequencing (Illumina). All 5376 variants were detected in the initial library and after the first cycle of sorting, indicating that the library coverage was complete. As the sorting cycles increased, some variants were no longer detected, and some sequences were enriched (Supplementary Table S3). The riboswitch variants were ranked according to their ‘enrichment trend’ (see Materials and Methods). The top 20 sequences were individually synthesized and assayed in bulk PURE*frex* reactions using the same GFP11/GFP1–10 reporter system employed in droplet sorting. As negative controls, we chose five variants that were ranked lower in the sequencing analysis. All of the top 20 variants clearly activated expression in response to histamine albeit to different degrees. In contrast, none of the five negative controls showed any response to histamine (Figure 4A).

We then fused these riboswitches to the full-length reporter gene eGFP (239 amino acids) because the above assay does not directly report the expression level of GFP11. Interestingly, the ON/OFF levels and ratios of the riboswitches varied greatly compared to the GFP11 assay (Figure 4B, C). For example, the ON/OFF ratio of the variant 19 (HA-C1g-19) was only 2.3 in the GFP11 assay but it was over 18 in the eGFP assay, mainly due to the higher ON level compared to the other variants. We speculate that the ON levels for some variants in the GFP11 assay were saturated due to the limiting concentration of the GFP1–10 fragment (approximately 26 μM) in the solution. This is also evident from the other switching variants and the no-riboswitch control (P_T7_-RBS-GFP11) exhibiting similarly high fluorescence levels at the ON-state in the GFP11 assay (Figure 4A). On the other hand, the OFF levels of the switches in the absence of histamine follow a similar trend in both GFP11 and eGFP assays (Figure 4A, C) because they are not likely to be affected by signal saturation. Moreover, we cannot exclude the possibility that the full-length mRNA may affect the riboswitch performance. Importantly, none of the negative controls exhibited any response to histamine in the eGFP assay.

**Figure 5.**
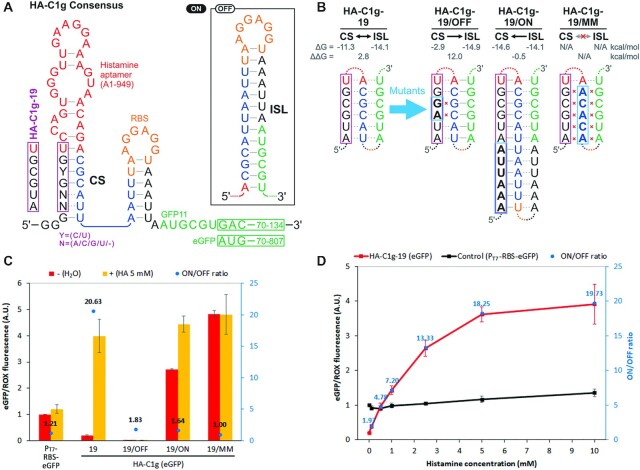
Characterization of the histamine ON-switches. (**A**) The consensus sequence of the enriched variants suggests formation of a competing stem (CS) in the presence of histamine resulting in destabilized inhibitory stem loop (ISL) to activate translation. (**B**) Sequences of HA-C1g-19 and its mutants. HA-C1g-19/OFF contains a double mutation that is designed to destabilize the CS. HA-C1g-19/ON contains an extended and stabilized CS. HA-C1g-19/MM was designed to disrupt both CS and ISL. The calculated free energy values (ΔG and ΔΔG) of the respective structures by ViennaRNA are shown above each sequence. (**C**) Histamine responses of HA-C1g-19 and its mutants fused to eGFP. The expression levels were normalized by the no-riboswitch control P_T7_-RBS-eGFP in the absence of histamine. (**D**) Dose-dependent expression of the HA-C1g-19 riboswitch (red) and the no-riboswitch control P_T7_-RBS-eGFP (black). The ON/OFF ratios were corrected for the nonspecific effects of histamine on P_T7_-RBS-eGFP at each concentration. Error bars represent the standard deviations of three independent assays. HA: histamine.

Inspection of the enriched variants confirms that these riboswitches most likely function by disrupting the ISL structure through forming a competing stem (CS) structure at the base of the aptamer (Figure 5A). All of the top 19 variants share the consensus sequence GYGU (Y = C or U) at the four nucleotides proximal to the aptamer (Figure 4C) that are likely to form a CS structure which is stabilized upon aptamer–ligand binding (Figure 5A). Consistent with this mechanism, introduction of mismatches within the randomized region that destabilize the CS in HA-C1g-19 resulted in low gene expression with or without histamine (HA-C1g-19/OFF, Figure 5B, C). Conversely, stabilization of the CS by increasing its length resulted in a higher expression level in the absence of histamine (HA-C1g-19/ON, Figure 5B, C). Finally, destabilization of both the ISL and the CS structures by mutating the 5′ end of the ISL resulted in constitutive expression as expected (HA-C1g-19/MM, Figure 5B, C).

Dose dependence analysis of HA-C1g-19-eGFP up to 10 mM histamine showed its ON/OFF ratio reaching 19.7 with an EC_50_ of approximately 1.6 mM (Figure 5D) after correcting for the moderate nonspecific activation of P_T7_-RBS-eGFP by histamine (up to 36% at 10 mM histamine) which does not contain the histamine aptamer. The higher concentration of the effector molecule necessary to activate the riboswitch (EC_50_) relative to the K_d_ of the aptamer (371 nM in this case) has been commonly observed in natural and synthetic riboswitches. These observations have generally been explained by the kinetic trapping mechanism in which switching of the mRNA folding in the ON state is determined cotranscriptionally (38,56–60). Interestingly, HA-C1g-19-eGFP shows a much higher ON level compared to the no-riboswitch control (P_T7_-RBS-eGFP) even though they share the same RBS sequence. We measured the kinetics of eGFP and mRNA levels of HA-C1g-19 and confirmed that the stronger signal is due to the higher translation efficiency in the presence of histamine, and that the mRNA level is not significantly affected by histamine (Supplementary Figure S5). The mechanistic basis of this unexpectedly high expression level remains to be investigated.

### Histamine OFF-riboswitches

We next sought to engineer histamine OFF-riboswitches that downregulate gene expression in response to histamine. OFF-riboswitches are relatively common in nature where riboswitches are often used to implement negative-feedback regulation of biosynthetic pathways. However, synthetic OFF-riboswitches in living cells are noticeably less common compared to ON-riboswitches. While natural and synthetic OFF-riboswitches in bacteria are expected to function in prokaryotic CFPS systems, to our knowledge, there are no previous reports of OFF-riboswitches implemented in prokaryotic CFPS systems (34).

**Figure 6.**
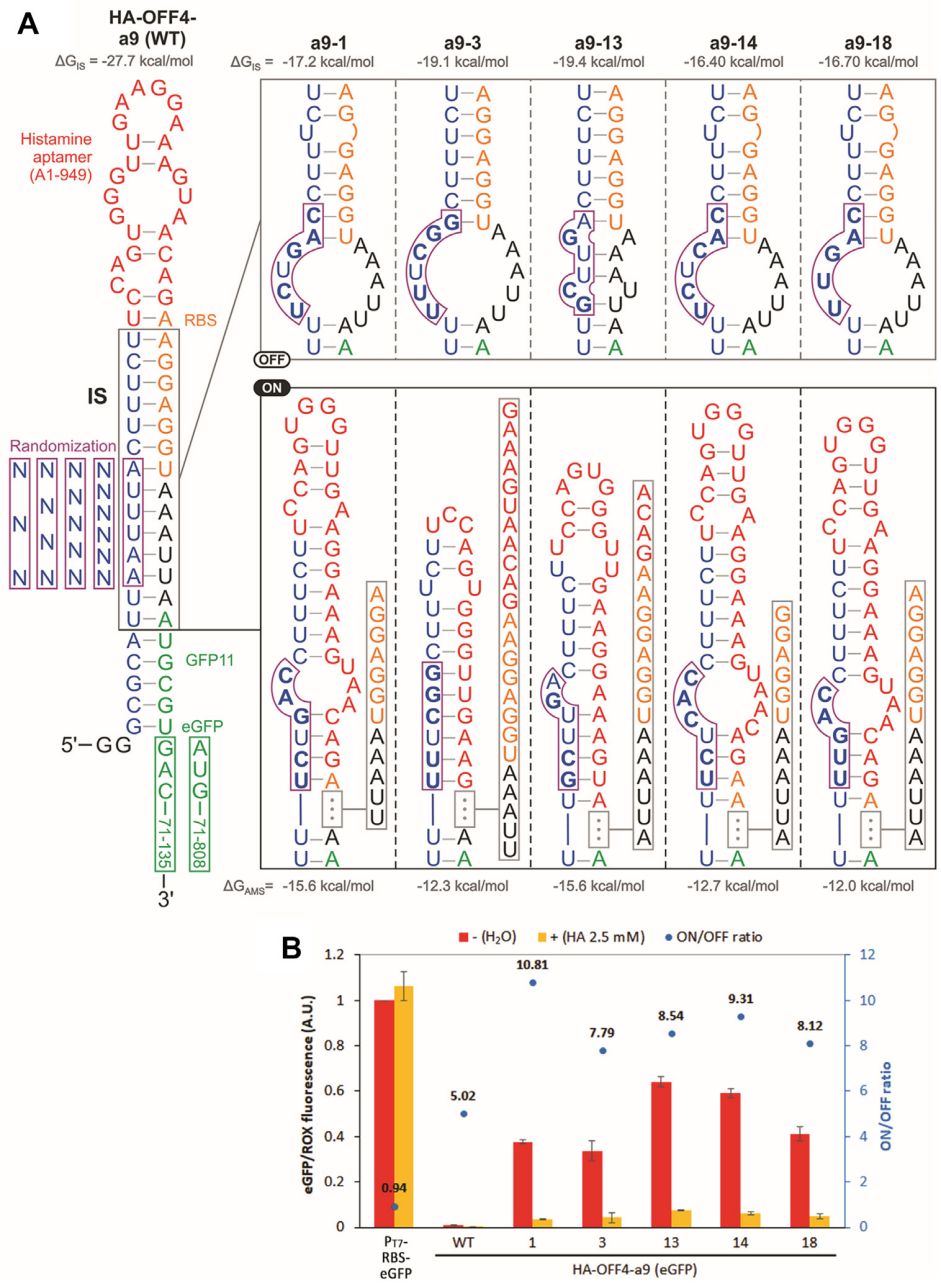
Histamine OFF-switches. (**A**) Riboswitch library design and the predicted OFF- and ON-structures of the variants discovered by droplet sorting. The library was based on HA-OFF4-a9 (WT) that is predicted to form an extended inhibitory stem (IS) structure sequestering the RBS and the start codon. The boxed nucleotides were replaced with random sequences. The variants discovered by droplet sorting are suggested to form an alternative metastable structure (AMS) that disrupts the RBS-sequestering stem (IS-like structure) in the absence of histamine. The RNA can adopt multiple metastable structures, and the diagram only shows those with the lowest folding energy. The calculated free energy values (ΔG_IS_ and ΔG_AMS_) of the respective structures by ViennaRNA are shown above or below each structure. (**B**) Individual characterization of the discovered variants using full-length eGFP as a reporter gene. The expression levels were normalized by the no-riboswitch control P_T7_-RBS-eGFP in the absence of histamine. The error bars represent standard deviations of three independent assays. HA: histamine.

We redesigned the histamine riboswitch library to increase the chances of discovering OFF-riboswitches. Our library was designed based on HA-OFF4-a9 that contains an extended inhibitory stem (IS) that blocks both RBS and the start codon with the histamine aptamer inserted in the loop region (Figure 6A). As expected, HA-OFF-a9 strongly represses gene expression with or without histamine (WT, Figure 6B). We randomized 3–6 bases upstream of the aptamer near the middle of the IS with an expectation that in the absence of histamine, some of the variants would form an alternative structure that disrupts the IS. We also expected that aptamer–ligand binding would restore the IS-like structure repressing translation (Figure 6A). This library (with up to 5440 variants) was subjected to alternating OFF-sorting in the presence of histamine (5 or 2.5 mM) and ON-sorting in the absence of histamine for a total of five rounds (three OFF-sorting and two ON-sorting) (Supplementary Figure S6A). Similar to the histamine ON-riboswitch sorting, we observed that the response to histamine of the bulk library population improved after the sorting (Supplementary Figure S6A).

After sequence analysis (Supplementary Figure S7B), we individually screened the top 20 variants by GFP11 assay (Supplementary Figure S8A). All variants were downregulated by histamine, with ON/OFF ratios ranging from 2.3 to 4.5. Ten variants were further screened by eGFP assay and all of them showed ON/OFF ratios over 6.1 (Supplementary Figure S8B). Detailed analysis of the best five variants confirmed ON/OFF ratios between 7.7 and 10.8 (Figure 6B). Interestingly, dose-dependent response of HA-OFF4-a9-13 showed a more sensitive response compared to the ON-riboswitch, with an EC_50_ of approximately 50 μM (Supplementary Figure S10).

Secondary structure predictions of the OFF-switches by ViennaRNA (49) web server suggest that the randomized region destabilizes the IS structure due to multiple mismatches within the IS, and it potentially allows an alternative metastable structure (AMS) to form by interacting with the aptamer sequence (Figure 6A). The AMS, presumably kinetically stabilized in the absence of histamine, makes the RBS and the start codon more accessible for the ribosome to initiate translation. Further mutational and kinetic analysis are necessary to validate this mechanistic hypothesis.

### Ciprofloxacin ON-riboswitches

To further demonstrate the generality of our droplet sorting strategy, we sought to develop cell-free riboswitches that respond to ciprofloxacin. Ciprofloxacin is a broad-spectrum fluoroquinolone antibiotic. Therefore, it is not amenable to the existing bacterial screening or selection methods unless fluoroquinolone-resistant strains are employed. Plasmid-mediated quinolone resistance (PMQR) mechanisms by themselves can only provide low-level resistance to ciprofloxacin (61), whereas higher-level resistance often requires multiple mutations in the genes targeted by the antibiotic (DNA gyrase *gyrA* and DNA topoisomerase IV *parC*) (62,63). Therefore, ciprofloxacin riboswitches represent another example where cell-free riboswitches cannot be easily transferred from bacterial riboswitches.

The Suess group discovered the first ciprofloxacin aptamers by *in vitro* selection (SELEX), and then used cellular screening in yeast to obtain riboswitches with ON/OFF ratios up to 7.5 (64). Another ciprofloxacin aptamer R10K6 (K_d_= 31 nM) was later identified which was used to engineer paper-based biosensors (65).

**Figure 7.**
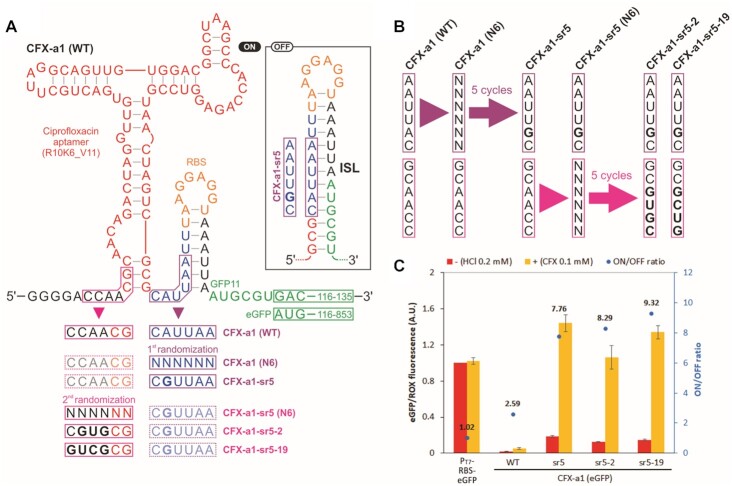
Ciprofloxacin ON-switches. (**A**) Riboswitch library design. Inhibitory stem loop (ISL) was designed to repress gene expression in the absence of histamine. Nucleotides in the boxed regions were randomized sequentially to discover variants that respond to ciprofloxacin. (**B**) Summary of the droplet sorting results. In the first round of sorting cycles, six nucleotides within the ISL of CFX-a1 (WT) were randomized to identify CFX-a1-sr5. In the second round of sorting cycles, six nucleotides upstream of CFX-a5-sr5 were randomized to isolate CFX-a5-sr5-2 and CFX-a5-sr5-19. (**C**) Individual characterization of the discovered variants using full-length eGFP as a reporter gene. The expression levels were normalized by the no-riboswitch control P_T7_-RBS-eGFP in the absence of ciprofloxacin. The error bars represent standard deviations of three independent replicates. CFX: ciprofloxacin.

We replaced the histamine aptamer in the histamine ON-riboswitch library described above with a shorter version of the R10K6 aptamer (R10K6_V11, K_d_= 36 nM, Figure 7A) (65). The resulting construct CFX-a1 exhibited attenuated expression in the presence and absence of ciprofloxacin (WT, Figure 7C). We speculated that the ISL was too stable (Figure 7A); therefore, we decided to randomize six nucleotides within the ISL to search for sufficiently destabilized ISL variants are still stable in the absence of ciprofloxacin but would be disrupted when the aptamer is bound to ciprofloxacin. Five cycles of sorting of this library (Figure 7B, Supplementary Figures S7C and S9A) resulted in CFX-a1-sr5 with an ON/OFF ratio of 7.8 (Figure 7C). We sought to further optimize the riboswitch performance by randomizing the six bases upstream of the aptamer with an expectation that these positions may further stabilize the aptamer-ligand structure and/or compete with and destabilize the ISL (Figure 7A). This second round of sorting cycles (Figure 7B, Supplementary Figure S7D, S9B, S9C) yielded CFX-a1-sr5-2 and CFX-a1-sr5-19 with moderately improved ON/OFF ratios of 8.3 and 9.3, respectively (Figure 7C). Dose-dependence analysis of the CFX-a1-sr5-19 riboswitch showed up to 9.8-fold activation with an EC_50_ of approximately 15 μM (Supplementary Figure S11) after correcting for nonspecific activation of gene expression caused at high concentrations of ciprofloxacin (up to 35% at 0.25 mM ciprofloxacin).

### Droplet-droplet communication using histamine riboswitch

Cell-free riboswitches enable a cell-free system to detect chemical signals from the environment (receiver) and to respond by modulating protein expression. If another cell-free system can synthesize the chemical signal (sender) and release it to the environment, the two systems can communicate, albeit only one-way, via a chemical signal. Previously, liposome-based artificial cells have been used to demonstrate chemical communication using a theophylline-responsive riboswitch (37,66). In these examples, the sender artificial cells contained theophylline-responsive riboswitches controlling the synthesis of α-hemolysin. Once theophylline is added to the medium, it triggers the formation of α-hemolysin pores on the lipid bilayer membrane allowing the release of a different signaling molecule (e.g. IPTG, doxycycline) trapped inside the artificial cell. In these examples, cell-free riboswitches were simply used as a trigger to release pre-encapsulated chemical signal molecules to the environment.

**Figure 8.**
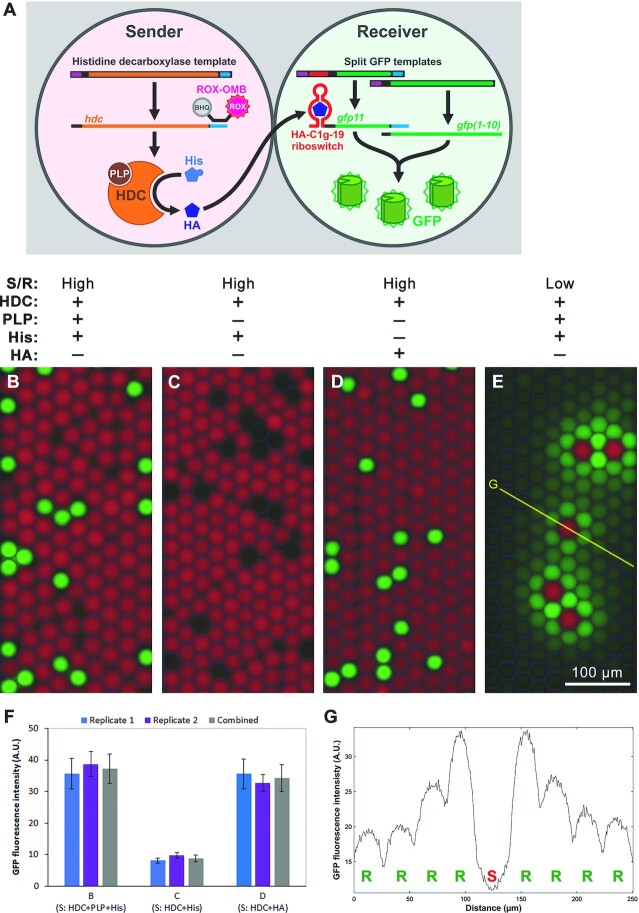
Chemical communication between droplets via histamine. (**A**) Overview of the chemically communicating droplets. The sender droplets (red) contain DNA templates that encode histidine decarboxylase (HDC) that converts L-histidine (His) into histamine (HA). Histamine synthesized by the sender droplets diffuses to the receiver droplets (green or non-fluorescent) which contains the histamine ON-riboswitch HA-C1g-19 controlling GFP11 expression, while the GFP1–10 fragment is constitutively expressed. ROX-OMB was included only in the sender droplets to distinguish the two species. (**B–E**) Fluorescence micrographs (merged: GFP + ROX + bright field) of the droplet populations. The droplets were prepared separately and mixed as described, then they were incubated at 37°C for 4 h. The same experiment was performed twice and yielded similar results. Contrast and brightness were enhanced uniformly for visualization. S/R: sender/receiver ratio. (**B**) The sender droplets in excess relative to the receiver droplets. (**C**) Same as **B** but without the essential HDC cofactor pyridoxal phosphate (PLP) in the sender droplets. (**D**) Same as **C** but histidine was substituted with histamine. (**E**) The receiver droplets in excess relative to the sender droplets. (**F**) GFP intensity of the receiver droplets calculated from the GFP channel of multiple fluorescence micrographs of conditions **B**, **C**, and **D** using ImageJ 1.52p. The experiment was performed twice yielding similar results. (**G**) GFP intensity profile of the cross-section (yellow line) of the GFP channel in panel **E** shows a fluorescence gradient in the receiver droplets (R) surrounding the sender droplet (S).

In this work, we attempted to use histamine as a chemical signal to mediate communication between droplets. The sender droplets contain a DNA template encoding histidine decarboxylase (HDC) that produces histamine from L-histidine using pyridoxal phosphate (PLP) as a cofactor. The receiver droplets contain a DNA template encoding GFP11 under the control of the histamine ON-riboswitch (HA-C1g-19) and another DNA template that constitutively expresses the GFP1–10 fragment (Figure 8A). When the sender and the receiver droplets were mixed and observed under a microscope, the receiver droplets that were surrounded by the sender droplets containing PLP (Figure 8B, F) clearly showed higher GFP fluorescence compared to those surrounded by the sender droplets without PLP (Figure 8C, F). When the receiver droplets were mixed in excess to the sender droplets, a spatial gradient of GFP expression in the receiver droplets was observed, with the droplets closer to the sender showing stronger GFP fluorescence (Figure 8E, G). The sender droplets containing only histamine confirm that the receiver signal is triggered by histamine, not PLP or L-histidine (Figure 8D). Separately, we confirmed that PLP is essential for histamine production by HDC expressed in PURE*frex* (Supplementary Figure S12), and that the histamine riboswitch is not appreciably activated by L-histidine or PLP (Supplementary Figure S13).

This artificial chemical communication mediated by histamine is analogous to bacterial cell-cell communication and quorum sensing systems in which the diffusive signals are produced by cells via an enzymatic reaction and then detected by the receiving cells using a chemical sensor (e.g. transcription factors) (67,68). Cell-free riboswitches may enable development of additional synthetic chemical communication systems for basic and applied research.

## DISCUSSION

Compared to the synthetic riboswitches that function in living cells, cell-free riboswitches suffer fewer biological constraints such as ligand toxicity and cell permeability. Since RNA aptamers can be selected *in vitro* to recognize diverse molecules, cell-free riboswitches may enable cell-free systems to detect and respond to a wide variety of chemical signals. In our recent work, we engineered cell-free riboswitches that respond to histamine which is not compatible with *E. coli* due to its low bioavailability (38). Therefore, we employed a semi-rational iterative design process which was slow and not generally applicable to other aptamer sequences. Several high-throughput screening or selection methods have been developed for engineering riboswitches in bacteria and have proven to be highly useful (41,42,44,56,69–72). However, riboswitches and other regulatory devices have not been engineered directly in cell-free systems in high-throughput. Low-throughput assays of individual devices in bulk solutions can also be very costly when using reconstituted CFPS systems. We addressed these challenges by developing a microfluidic droplet sorting strategy to rapidly enrich functional cell-free riboswitches from > 5000 variants.

The throughput of our method is currently limited by that of the droplet sorter which is approximately 6.3 × 10^5^ droplets/h. Due to the requirement of maintaining ∼10–30% magnetic bead encapsulation rate to ensure that the majority of the droplets contain single variant per droplet, the effective throughput decreases to ∼6 × 10^4^–1.9 × 10^5^ droplets/h. Nevertheless, 1–6 h of sorting can achieve sufficient coverage (10–40 ×) of a library with 6 randomized nucleotides (4096 variants). While this has been sufficient for our riboswitch libraries, higher throughput may be desirable for more complex systems, for example, Boolean logic gate riboswitches (73).

It should be noted that we used a commercially available droplet generator, droplet sorter, and microfluidic chips (see Materials and Methods), demonstrating that microfluidics-based screening in a CFPS system can be performed without specialized equipment and expertise. Such general-purpose microfluidics platforms are becoming increasingly accessible. Of course, further improvements in throughput and efficiency may be possible by adapting specialized techniques such as pico-injection (74) and multichannel sorting (75) that are yet to be commoditized.

Proof-of-principle sorting of enzymes expressed in CFPS system-containing monodisperse droplets has been demonstrated multiple times (76,77), but high-throughput screening from a randomized protein library was only recently achieved by Holstein et al. who evolved the serine protease Savinase mutants with improved activity (78). Alternatively, Zhang et al. developed femtoliter droplet arrays in which enzymes are expressed in the PURE system from single DNA templates (79). They achieved 20-fold improvement of alkaline phosphatase activity after screening. It should be noted that while microfluidic droplet screening of RNA devices such as aptamers (80) and ribozymes (81) have been reported, none of them are based on CFPS systems.

To our knowledge, there have been no attempts to engineer dynamic biomolecular devices such as riboswitches directly in CFPS systems in high-throughput. One of the technical challenges in screening genetic devices or circuits in droplet-based methods is the efficiency of OFF-sorting. While it is straightforward to sort for ON-droplets using a fluorescence reporter, isolating rare OFF-droplets is complicated by the large proportion of empty droplets without DNA template. We avoided sorting empty droplets during OFF-sorting cycles by using a molecular beacon as a reporter of mRNA transcription. This strategy should also be useful for high-throughput engineering of protein-based switches in CFPS systems. Current efforts to build sophisticated cell-free systems such as artificial cells and biosensors mostly rely on parts and devices borrowed from or engineered in living cells. Our work should pave the way for efficient engineering of cell-free genetic devices directly in CFPS systems optimized for cell-free applications.

## CONCLUSIONS

We developed a high-throughput screening method for riboswitches directly in a cell-free system. The droplet sorting method is straightforward and does not require specially customized instrumentation. The method was used to develop three types of cell-free riboswitches that respond to two small molecules. A histamine-responsive riboswitch was used to demonstrate artificial chemical communication between droplets that mimic bacterial quorum sensing systems. Our droplet sorting strategy should greatly facilitate cell-free riboswitch development which in turn can diversify the chemical interface available to cell-free systems such as artificial cells.

## DATA AVAILABILITY

Nucleic acid sequences, numerical data for Figures 4A, B, 5C, D, 6B and 7C are provided as an Excel file in Supplementary Data. All data and materials are available upon request.

## Supplementary Material

gkac152_Supplemental_Files
