# Supplementary material for: High-throughput screening of cell-free riboswitches by fluorescence-activated droplet sorting
Source: Nucleic Acids Res. 2022 Mar 7;50(6):3535–50. doi: 10.1093/nar/gkac152 (PMC8989549; doi:10.1093/nar/gkac152)
Supplement: gkac152_Supplemental_Files [file gkac152_supplemental_files.zip › Tabuchi_NAR_supp_info_1.pdf]

Supplementary Information

High-throughput screening of cell-free riboswitches by fluorescence-activated droplet sorting

Takeshi Tabuchi and Yohei Yokobayashi*

Nucleic Acid Chemistry and Engineering Unit

Okinawa Institute of Science and Technology Graduate University

Onna, Okinawa 904-0495, Japan

*Corresponding author: Yohei Yokobayashi

Email: yohei.yokobayashi@oist.jp

Supplementary Table S1. List of primers and oligonucleotides.

Name	Sequence
P0L (Anchor)	(5′ Dual Biotin + iSp18)TggcgaaaggtgcttgttgTAATACGACTC
P3L (Forward)	ggcgaaaggtgcttgttgTAATACGACTC
P4L (Reverse)	(5′ Texas Red)tagtacgatgccagcaggtcTTAtgtaatcc
P-eGFP-R	tagtacgatgccagcaggtcTCActtg
ROX-OMB (2′O-methyl-RNA)	(5′ ROX)mCmCmUmGmUmAmCmGmAmUmGmCmCmAmGmCmAmGmG(3′ BHQ2)
P-HA-Nova-T1	acactctttccctacacgacgctcttccgatct(Custom_Barcode)ggcgaaaggtgct tgttgtaatacg
P-HA-Nova-B1	gtgactggagttcagacgtgtgctcttccgatct(Custom_Barcode)aggaccatgtgg tcacgcat
TruSeq-i5 (UDI00##)	aatgatacggcgaccaccgagatctacac(UDI00##)acactctttccctacacgacgc
TruSeq-i7 (UDI00##)	caagcagaagacggcatacgagat(UDI00##)gtgactggagttcagacgtgtg

Supplementary Table S2. Sequences of the constructs described in this work. To keep this table concise, only the variable or unique sequences are shown, while the shared constant regions have been abbreviated as Leader (5' end) and Trailer (3' end). All GFP11 constructs have the structure: (5') Leader #1…Variable Sequence…Trailer #1 (3'). All eGFP constructs have the structure: (5') Leader #2…Variable Sequence…Trailer #2 (3'). Tag 1 and tag 2 sequences are underlined. The T7 promoter is shown in UPPERCASE. Aptamers are shown in **BOLD UPPERCASE** letters. The coding sequences (ORFs) are shown in *italicized lowercase* letters. The start and the stop codons are shown in *ITALICIZED UPPERCASE* letters. Restriction sites are shown in **bold lowercase** letters.

Description	Sequence (5'-3')
Leader #1 (tag 1 + P_{T7})	ggcgaaaggtgcttgttgTAATACGACTCACTATAg…
Trailer #1 (*gfp11* + tag 2)	…*ATGcgtgaccacatggtccttcatgagtatgtaaatgctgctgggattacaTAA*gacctgctggcatcgtacta
Leader #2 (P_{T7})	ttgTAATACGACTCACTATAg…
Trailer #2 (*egfp* + tag 2)	…*ATGcgtATGgtgagcaagggcgaggagctgttcaccggggtggtgcccatcctggtcgagctggacggcgacgtaaacggccacaagttcagcgtgtccggcgagggcgagggcgatgccacctacggcaagctgaccctgaagttcatctgcaccaccggcaagctgcccgtgccctggcccaccctcgtgaccaccctgacctacggcgtgcagtgcttcagccgctaccccgaccacatgaagcagcacgacttcttcaagtccgccatgcccgaaggctacgtccaggagcgcaccatcttcttcaaggacgacggcaactacaagacccgcgccgaggtgaagttcgagggcgacaccctggtgaaccgcatcgagctgaagggcatcgacttcaaggaggacggcaacatcctggggcacaagctggagtacaactacaacagccacaacgtctatatcatggccgacaagcagaagaacggcatcaaggtgaacttcaagatccgccacaacatcgaggacggcagcgtgcagctcgccgaccactaccagcagaacacccccatcggcgacggccccgtgctgctgcccgacaaccactacctgagcacccagtccgccctgagcaaagaccccaacgagaagcgcgatcacatggtcctgctggagttcgtgaccgccgccgggatcactctcggcatggacgagctgtacaagTGA*gacctgctggcatcgtacta
P_{T7}-RBS-GFP11 (Strong RBS)	(Leader #1)…**gaattc**taaggaggtaaatta…(Trailer #1)
P_{T7}-wRBS-GFP11 (Weak RBS)	(Leader #1)…**gagctc**tcacacaggac…(Trailer #1)
P_{T7}-RBS-eGFP	(Leader #2)…ggttaaggaggtaaatta…(Trailer #2)
HA-C1g (N6)	(Leader #1/#2)…ggNNNNN**NCCAGTGGGTTGAAGGAAAGTAACAGA**cgcattaatttaaggaggtaaatta…(Trailer #1/#2)
HA-C1g (N5)	(Leader #1/#2)…ggNNNN**NCCAGTGGGTTGAAGGAAAGTAACAGA**cgcattaatttaaggaggtaaatta…(Trailer #1/#2)
HA-C1g (N4)	(Leader #1/#2)…ggNNN**NCCAGTGGGTTGAAGGAAAGTAACAGA**cgcattaatttaaggaggtaaatta…(Trailer #1/#2)
HA-C1g-19	(Leader #1/#2)…ggatgcg**TCCAGTGGGTTGAAGGAAAGTAACAGA**cgcattaatttaaggaggtaaatta…(Trailer #1/#2)
HA-C1g-19/OFF	(Leader #1/#2)…ggatagg**TCCAGTGGGTTGAAGGAAAGTAACAGA**cgcattaatttaaggaggtaaatta…(Trailer #1/#2)
HA-C1g-19/ON	(Leader #1/#2)…ggaaattaatgcg**TCCAGTGGGTTGAAGGAAAGTAACAGA**cgcattaatttaaggaggtaaatta…(Trailer #1/#2)
HA-C1g-19/MM	(Leader #1/#2)…ggatgcg**TCCAGTGGGTTGAAGGAAAGTAACAGA**acacataatttaaggaggtaaatta…(Trailer #1/#2)
HA-OFF4-a9 (WT)	(Leader #1/#2)…ggcgcattaatttactttct**TCCAGTGGGTTGAAGGAAAGTAACAGA**aggaggtaaatta…(Trailer #1/#2)
HA-OFF4-a9 (N6)	(Leader #1/#2)…ggcgcattNNNNNNctttct**TCCAGTGGGTTGAAGGAAAGTAACAGA**aggaggtaaatta…(Trailer #1/#2)
HA-OFF4-a9 (N5)	(Leader #1/#2)…ggcgcattNNNNNctttct**TCCAGTGGGTTGAAGGAAAGTAACAGA**aggaggtaaatta…(Trailer #1/#2)
HA-OFF4-a9 (N4)	(Leader #1/#2)…ggcgcattNNNNctttct**TCCAGTGGGTTGAAGGAAAGTAACAGA**aggaggtaaatta…(Trailer #1/#2)
HA-OFF4-a9 (N3)	(Leader #1/#2)…ggcgcattNNNctttct**TCCAGTGGGTTGAAGGAAAGTAACAGA**aggaggtaaatta…(Trailer #1/#2)
HA-OFF4-a9-1	(Leader #1/#2)…ggcgcatttctgacctttct**TCCAGTGGGTTGAAGGAAAGTAACAGA**aggaggtaaatta…(Trailer #1/#2)
HA-OFF4-a9-3	(Leader #1/#2)…ggcgcattttcggctttct**TCCAGTGGGTTGAAGGAAAGTAACAGA**aggaggtaaatta…(Trailer #1/#2)
HA-OFF4-a9-13	(Leader #1/#2)…ggcgcattgcttgactttct**TCCAGTGGGTTGAAGGAAAGTAACAGA**aggaggtaaatta…(Trailer #1/#2)
HA-OFF4-a9-14	(Leader #1/#2)…ggcgcatttctcacctttct**TCCAGTGGGTTGAAGGAAAGTAACAGA**aggaggtaaatta…(Trailer #1/#2)
HA-OFF4-a9-18	(Leader #1/#2)…ggcgcattttgacctttct**TCCAGTGGGTTGAAGGAAAGTAACAGA**aggaggtaaatta…(Trailer #1/#2)
CFX-a1 (WT)	(Leader #1/#2)…gggaccaa**CGCAACAGACTAGGTTGTGACTGCTTAGGCAGTTGTGGACGGCTAAGCCCACCAGAGGTCCGTAACTAGTCG**cgcattaatttaaggaggtaaatta…(Trailer #1/#2)
CFX-a1 (N6)	(Leader #1/#2)…gggaccaa**CGCAACAGACTAGGTTGTGACTGCTTAGGCAGTTGTGGACGGCTAAGCCCACCAGAGGTCCGTAACTAGTCG**cgNNNNNNtttaaggaggtaaatta…(Trailer #1/#2)

CFX-a1-sr5	(Leader #1/#2)…gggaccaa**CGCAACAGACTAGGTTGTGACTGCTTAGGCAGTTGTGGACGGCTAAGCCC**<br>**ACCAGAGGTCCGTAACTAGTCG**cgcgttaatttaaggaggtaaatta…(Trailer #1/#2)
CFX-a1-sr5 (N6)	(Leader #1/#2)…gggaNNNN**NNCAACAGACTAGGTTGTGACTGCTTAGGCAGTTGTGGACGGCTAAGCCC**<br>**ACCAGAGGTCCGTAACTAGTCG**cgcgttaatttaaggaggtaaatta…(Trailer #1/#2)
CFX-a1-sr5-2	(Leader #1/#2)…gggacgtg**CGCAACAGACTAGGTTGTGACTGCTTAGGCAGTTGTGGACGGCTAAGCCC**<br>**ACCAGAGGTCCGTAACTAGTCG**cgcgttaatttaaggaggtaaatta…(Trailer #1/#2)
CFX-a1-sr5-19	(Leader #1/#2)…gggagtcg**CGCAACAGACTAGGTTGTGACTGCTTAGGCAGTTGTGGACGGCTAAGCCC**<br>**ACCAGAGGTCCGTAACTAGTCG**cgcgttaatttaaggaggtaaatta…(Trailer #1/#2)
P_{T7}-RBS-GFP(1-10)	tgattgTAATACGACTCACTATAgggaacagaaggaggtaaatta*ATGcgtatgagcaaaggagaagaacttt*<br>*tcactggagttgtcccaattcttgttgaattagatggtgatgttaatgggcacaaattttctgtcagaggaga*<br>*gggtgaaggtgatgctacaatcggaaaactcacccttaaatttatttgcactactggaaaactacctgttcca*<br>*tggccaacacttgtcactactctgacctatggtgttcaatgcttttcccgttatccggatcacatgaaaaggc*<br>*atgactttttcaagagtgccatgcccgaaggttatgtacaggaacgcactatatctttcaaagatgacgggaa*<br>*atacaagacgcgtgctgtagtcaagtttgaaggtgatacccttgttaatcgtatcgagttaaagggtactgat*<br>*tttaaagaagatggaaacattctcggacacaaacttgagtacaactttaactcacacaatgtatacatcacgg*<br>*cagacaaacaaaagaatggaatcaaagctaacttcacagttcgccacaacgttgaagatggttccgttcaact*<br>*agcagaccattatcaacaaaatactccaattggcgatggccctgtccttttaccagacaaccattacctgtcg*<br>*acacaaactgtcctttcgaaagatcccaacgaaaagTccggcggtggaggtctcgagcatcatcatcatcatc*<br>*atTGA*gtttaaacggtctccagcttggc
P_{T7}-*hdc*-6xHis	ggcgaaaggtgcttgttgTAATACGACTCACTATAgggagatttgtttaactttaaggaggtaattttt*ATGa*<br>*cattatcaatctcagatcagaacaagttagatagcttctggtcatattgtgtaaagaaccagtattttaatat*<br>*cggataccctgaatccgcggattttgattatactattctggagcgctttatgcgtttcagtattaataactgc*<br>*ggtgactggggcgagtattgcaactaccttcttaattctttcgattttgagaaggaggttatggagtatttcg*<br>*cacaattattcaagattccattcgaggagtcctggggttacgtaaccaatggtggtacggaaggcaatatgtt*<br>*cggatgttacctgggacgtgagatcttccctaatggaactctttactacagcaaagacacccattacagcgta*<br>*gcaaaaattgtcaaactcttacgtattaagtccacgttggtcgagagccagcctaacggtgagatggattatg*<br>*ccgaccttatcaagaagatcaaggcagacaacgaaaaacaccctattatcttcgccaatatcggaaccactgt*<br>*gcgtggagcaattgacaatattgcgattattcagcaatcgatttcggaactgggaatcgaacgtaaggattac*<br>*tatttacacgcggatgccgcattgtcagggatgattcttccatttgtggataaccctcaaccgtttaatttcg*<br>*cggacgggattgatagtatcggagtctctgggcataagatgatcggaagtcccattccttgcgggattgtcgt*<br>*cgctaaaaagaagaatgtagatcgtatttccgttgaaatcgattacatttccgcgcatgacaaaacgatctca*<br>*ggatcgcgcaatggtcataccccattaatgatgtgggaagcaatccgttctcatagttgggaagaatggcgtc*<br>*gtcgcattgagcgttctttaaacatggcgcaatatgcagtggatcgtttccaaagcgcgggaattgatgcctg*<br>*gcgcaataagaactcgattacggttgtatttccttgcccttccgaggcggtttggaaaaagcactgcttggcg*<br>*acatctggagatatcgctcatcttatcgcaacggctcaccaccttgattcgtccaagatcgatgctttaatcg*<br>*acgatgtgattgcggacttgaagaaacaagctgcctcaggtggtggcggattagaacaccaccaccatcacca*<br>*tTAA*tggattacataagacctgctggcatcgtacta
P_{trc}-*gfp(1-10)*-6xHis (pTrcHis vector)	(…) CTGTTGACAATTAATCATCCGGCTCGTATAATGTGTGGAATTGTGAGCGGATAACAATTgaattaagctt<br>taaggaggatttagt*ATGagcaaaggagaagaacttttcactggagttgtcccaattcttgttgaattagatg*<br>*gtgatgttaatgggcacaaattttctgtcagaggagagggtgaaggtgatgctacaatcggaaaactcaccct*<br>*taaatttatttgcactactggaaaactacctgttccatggccaacacttgtcactactctgacctatggtgtt*<br>*caatgcttttcccgttatccggatcacatgaaaaggcatgactttttcaagagtgccatgcccgaaggttatg*<br>*tacaggaacgcactatatctttcaaagatgacgggaaatacaagacgcgtgctgtagtcaagtttgaaggtga*<br>*tacccttgttaatcgtatcgagttaaagggtactgattttaaagaagatggaaacattctcggacacaaactt*<br>*gagtacaactttaactcacacaatgtatacatcacggcagacaaacaaaagaatggaatcaaagctaacttca*<br>*cagttcgccacaacgttgaagatggttccgttcaactagcagaccattatcaacaaaatactccaattggcga*<br>*tggccctgtccttttaccagacaaccattacctgtcgacacaaactgtcctttcgaaagatcccaacgaaaag*<br>*tccggcggtggaggtctcgagcatcatcatcatcatcatTGA*(…)

Supplementary Table S3. Summary of sequencing statistics. max.: maximum, avg.: average, mdn.: median.

HA-C1g (N4,N5,N6)	
**Sequencing platform**	Illumina MiSeq v3
**Read ends**	Single-read
**Read length**	150 bp
**Run statistics**	
**Density (K/mm^2)**	1127 ± 42
**PF Clusters (M)**	25.97
**% PF Clusters**	93.43
**Yield (G)**	3.9
**% >= Q30 bases**	94.08
**Mean Quality**	36.28

Sequencing data						
	**Total**	**Cycle 0**	**Cycle 1**	**Cycle 2**	**Cycle 3**	**Cycle 4**
**Total raw reads**	22813551					
**Sorted reads**	22499883	11178476	5709002	3279952	1553967	778486
**Processed reads**	14819186	6713618	3863911	2436060	1181679	623918
**Unique variants (>0 reads)**	5376	5376	5376	5374	5341	5167
		100.0%	100.0%	100.0%	99.3%	96.1%
**Unique variants (>0.01% cycle reads)**		3854	3166	3206	2220	1953
		71.7%	58.9%	59.6%	41.3%	36.3%
**Reads per variant (max.)**		8759	14420	11192	41091	17426
**Reads per variant (avg.)**		1249	719	453	220	116
**Reads per variant (mdn.)**		995	480	316	80	22

HA-OFF4-a9 (N3,N4,N5,N6)	
**Sequencing platform**	Illumina NovaSeq 6000
**Read ends**	Paired-end
**Read length**	150 bp
**Run statistics**	
**% of lane**	12.40
**PF Clusters (M)**	60.97
**% PF Clusters**	100.0
**Yield (G)**	18.4
**% >= Q30 bases**	94.97
**Mean Quality**	36.16

Sequencing data							
	**Total**	**Cycle 0**	**Cycle 1**	**Cycle 2**	**Cycle 3**	**Cycle 4**	**Cycle 5**
**Total raw reads**	60973370						
**Sorted reads**	59511474	9197153	10207304	9940022	10067480	10016114	10083401
**Processed reads**	56590426	8693158	9820521	9534414	9588368	9528074	9425891
**Unique variants (>0 reads)**	5440	5440	5440	5440	5440	5440	5440
		100.0%	100.0%	100.0%	100.0%	100.0%	100.0%
**Unique variants (>0.01% cycle reads)**		4938	2179	2268	1532	1396	1065
		90.8%	40.1%	41.7%	28.2%	25.7%	19.6%
**Reads per variant (max.)**		10728	23539	22690	61720	88566	183589
**Reads per variant (avg.)**		1598	1805	1753	1763	1751	1733
**Reads per variant (mdn.)**		1464	282	335	74	68	71

CFX-a1 (N6)	
**Sequencing platform**	Illumina NovaSeq 6000
**Read ends**	Paired-end
**Read length**	150 bp
**Run statistics**	
**% of lane**	9.22
**PF Clusters (M)**	51.15
**% PF Clusters**	100.0
**Yield (G)**	15.4
**% >= Q30 bases**	96.40
**Mean Quality**	36.40

Sequencing data							
	**Total**	**Cycle 0**	**Cycle 1**	**Cycle 2**	**Cycle 3**	**Cycle 4**	**Cycle 5**
**Total raw reads**	50618895						
**Sorted reads**	50056790	12678321	4525667	4217190	17091491	4419733	7124388
**Processed reads**	35427450	11271195	3519599	3414738	10092592	3385559	3743767
**Unique variants (>0 reads)**	4096	4096	4096	4096	4096	4096	4096
		100.0%	100.0%	100.0%	100.0%	100.0%	100.0%
**Unique variants (>0.01% cycle reads)**		3579	1026	1123	718	658	632
		87.4%	25.0%	27.4%	17.5%	16.1%	15.4%
**Reads per variant (max.)**		41824	49916	34033	363582	196501	165315
**Reads per variant (avg.)**		2752	859	834	2464	827	914
**Reads per variant (mdn.)**		2254	89	64	204	56	85

CFX-a1-sr5 (N6)	
**Sequencing platform**	Illumina NovaSeq 6000
**Read ends**	Paired-end
**Read length**	150 bp
**Run statistics**	
**% of lane**	11.05
**PF Clusters (M)**	54.34
**% PF Clusters**	100.0
**Yield (G)**	16.4
**% >= Q30 bases**	95.26
**Mean Quality**	36.19

Sequencing data							
	**Total**	**Cycle 0**	**Cycle 1**	**Cycle 2**	**Cycle 3**	**Cycle 4**	**Cycle 5**
**Total raw reads**	54340974						
**Sorted reads**	45113130	9495092	8770285	9265975	5799156	5788918	5993704
**Processed reads**	41796625	8858803	8131638	8604911	5302821	5381831	5516621
**Unique variants (>0 reads)**	4096	4096	4096	4093	4090	4093	4093
		100.0%	100.0%	99.9%	99.9%	99.9%	99.9%
**Unique variants (>0.01% cycle reads)**		3223	2012	1891	1509	949	906
		78.7%	49.1%	46.2%	36.8%	23.2%	22.1%
**Reads per variant (max.)**		16169	25619	46445	48031	106391	163758
**Reads per variant (avg.)**		2163	1985	2101	1295	1314	1347
**Reads per variant (mdn.)**		1675	763	627	75	61	64

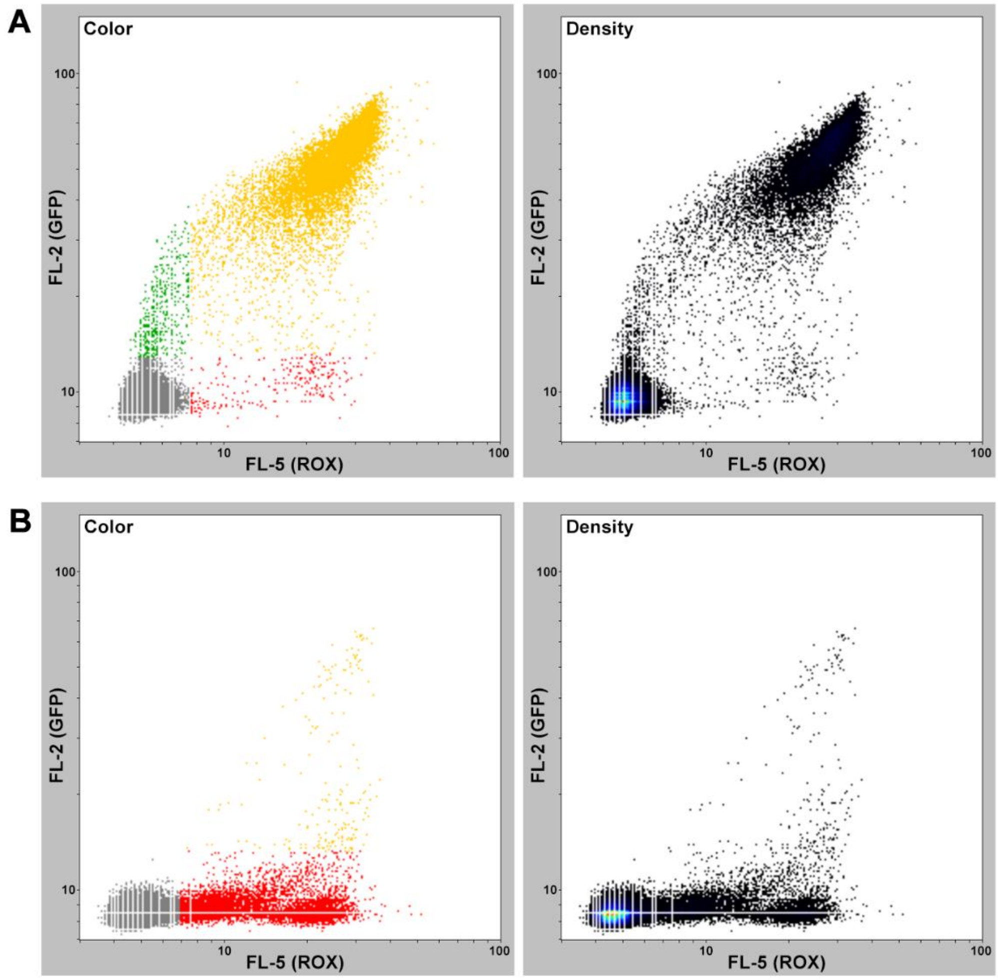

Supplementary Figure S1. Examples of the dual color fluorescence density plots of the droplet sorting experiments. The vertical axis (log scale) corresponds to GFP fluorescence (FL-2 channel), and the horizontal axis (log scale) corresponds to ROX fluorescence (FL-5 channel). Three different populations can be observed: empty droplets (grey: ROX-/GFP-, bottom left), OFF-droplets (red: ROX+/GFP-, bottom right), and ON-droplets (yellow/green: ROX+/GFP+, top right). (**A**) A population with high abundance of ON-droplets from the mock sorting experiment. (**B**) A population with high abundance of OFF-droplets from the mock sorting experiment.

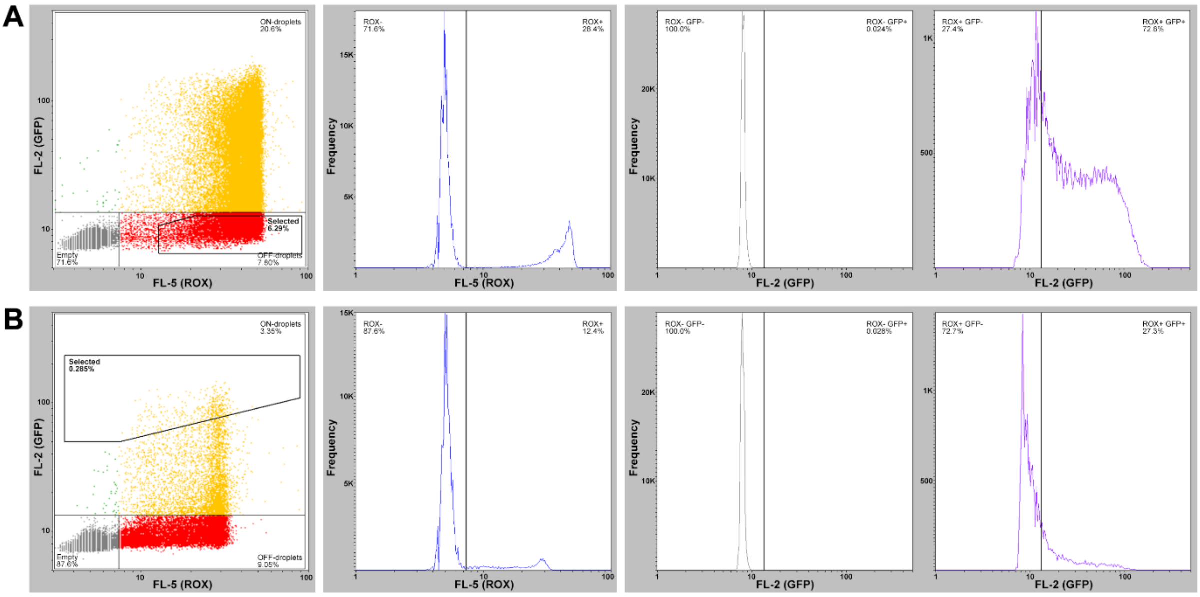

Supplementary Figure S2. Examples of population thresholds and selection gates used during the (**A**) OFF-droplet and (**B**) ON-droplet sorting. The example plots presented here correspond to the cycle 2 and 3 of the histamine ON-switch selection process. The gates labeled "Selected" (thicker border) correspond to the chosen selection gates. In the 2D plots, the vertical axis (log scale) corresponds to GFP fluorescence (FL-2 channel), and the horizontal axis (log scale) corresponds to ROX fluorescence (FL-5 channel). In the histograms, the vertical axis (linear scale) corresponds to the frequency, and the horizontal axis (log scale) corresponds to the respective fluorescence channel (FL-2 or FL-5 channels).

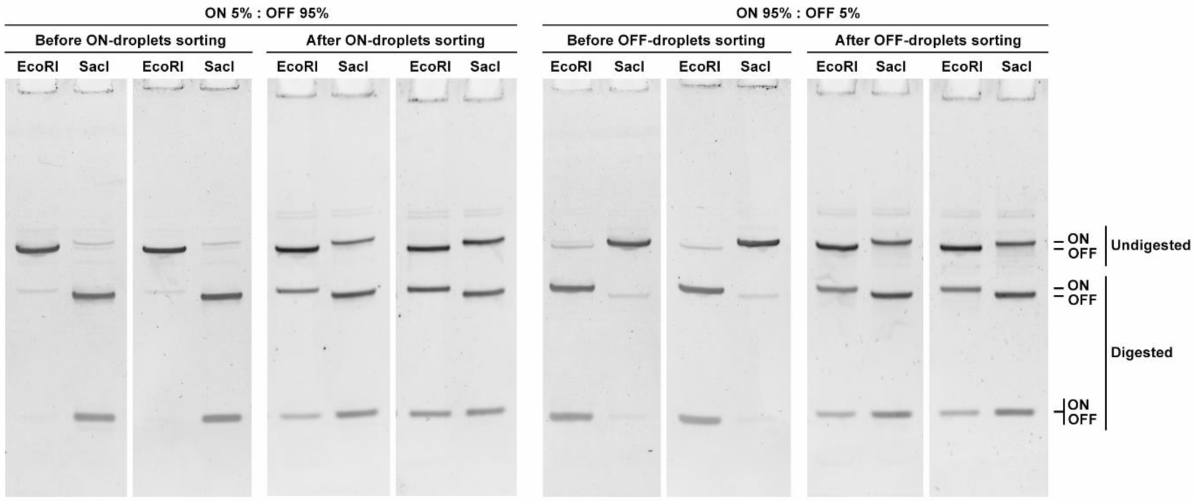

Supplementary Figure S3. Native PAGE (8%) of the DNA templates recovered from mock sorting after restriction digestion. The gels were stained by SYBR Gold. The ON templates are digested by EcoRI and the OFF templates are digested by SacI.

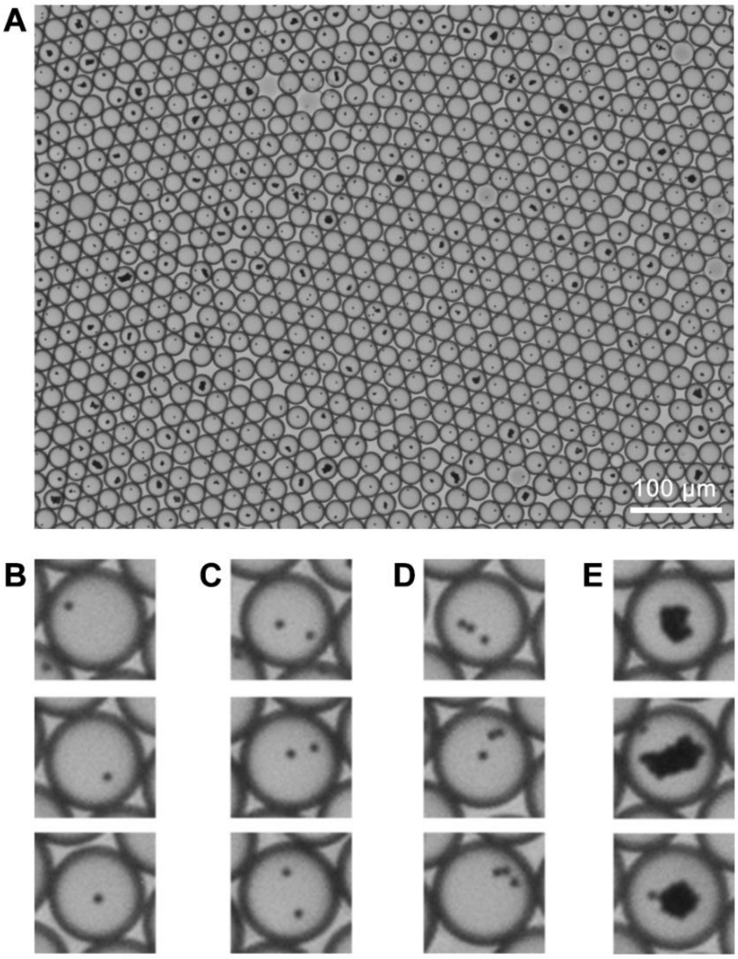

Supplementary Figure S4. An example of aggregation of the magnetic beads inside droplets. (**A**) Droplets recovered after ON-selection (mock sorting experiment) viewed under a light microscope (bright field). Magnification of the droplets containing (**B**) a single bead, (**C**) two beads, (**D**) multiple isolated beads, and (**E**) aggregated beads.

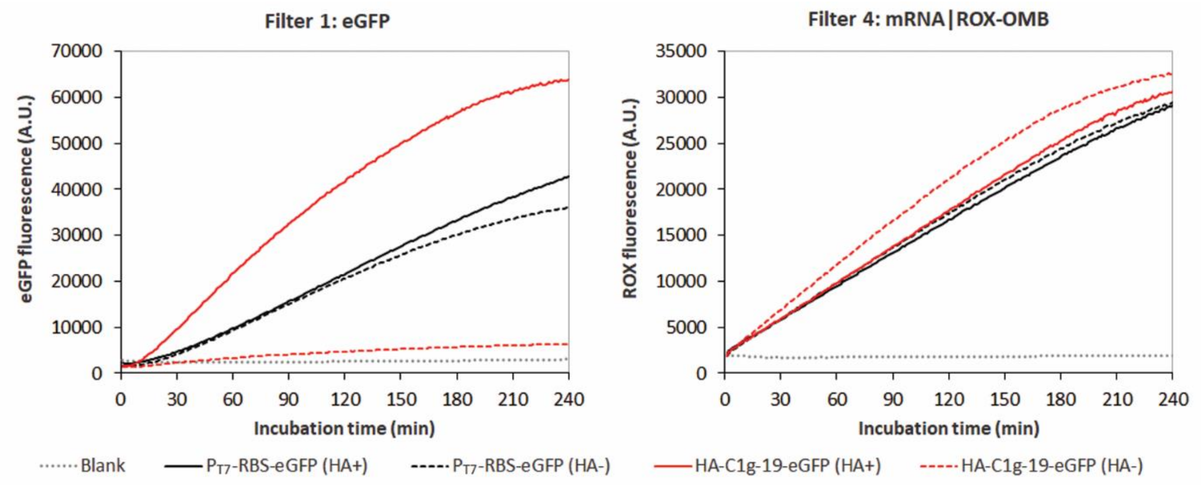

Supplementary Figure S5. Kinetics of eGFP (left) and mRNA (right) synthesis of the no-riboswitch control (P_{T7}-RBS-eGFP, black line) and HA-C1g-19-eGFP (red line) in a PURE*frex* 1.0 reaction with 2.5 µM of ROX-OMB. Blank: PURE*frex* reaction without DNA template (grey dotted line); HA+: 5 mM histamine (solid line); HA-: no histamine (dashed line). A substantial increase (~2-fold) in eGFP signal of HA-C1g-19-eGFP was observed during the course of the reaction compared to the P_{T7}-RBS-eGFP control. The graphs are based on average values of 2 replicate reactions. The incubation (37°C for 4 h) and real-time fluorescence measurements were performed in a Step One Plus Real-Time PCR System (Applied Biosystems) using filter 1 (GFP/FAM) and filter 4 (ROX).

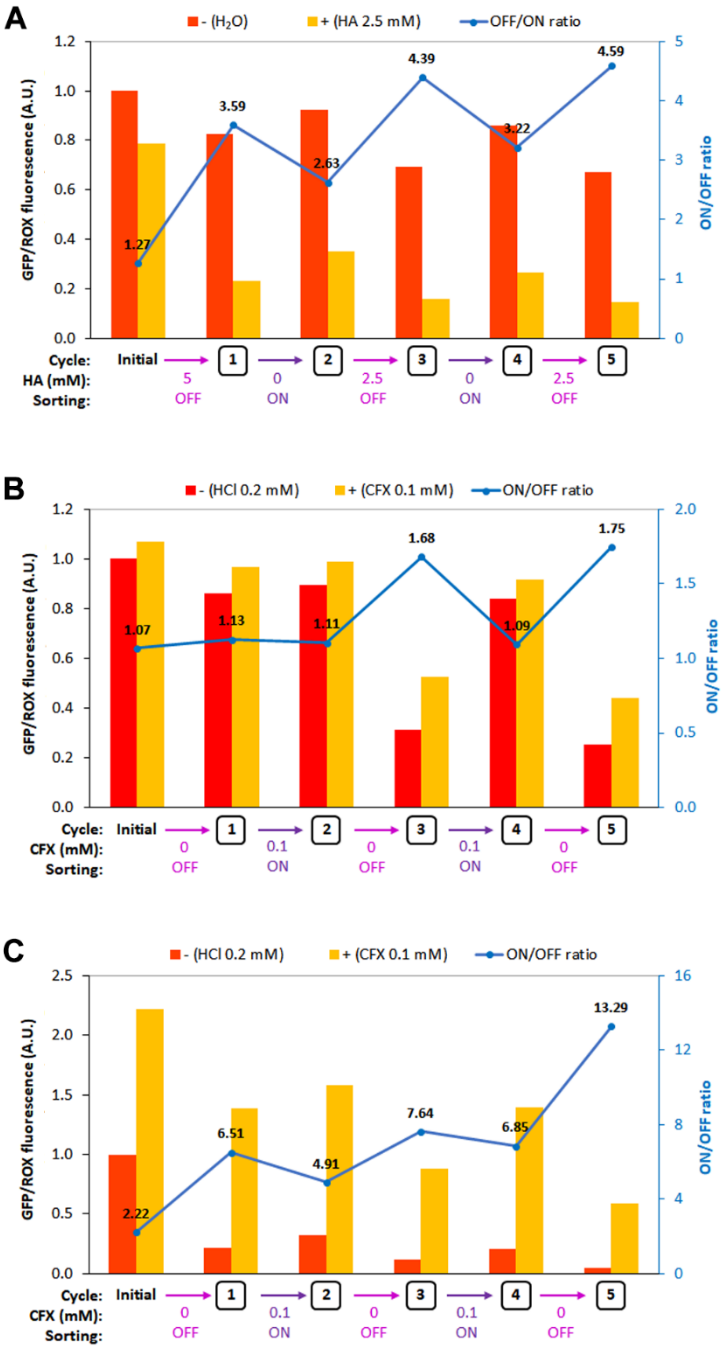

Supplementary Figure S6. Responses of the bulk riboswitch populations from different sorting cycles. (**A**) Histamine OFF-riboswitch library. (**B**) Ciprofloxacin ON-riboswitch library based on CFX-a1. (**C**) Ciprofloxacin ON-riboswitch library based on CFX-a1-sr5. HA: histamine, CFX: ciprofloxacin.

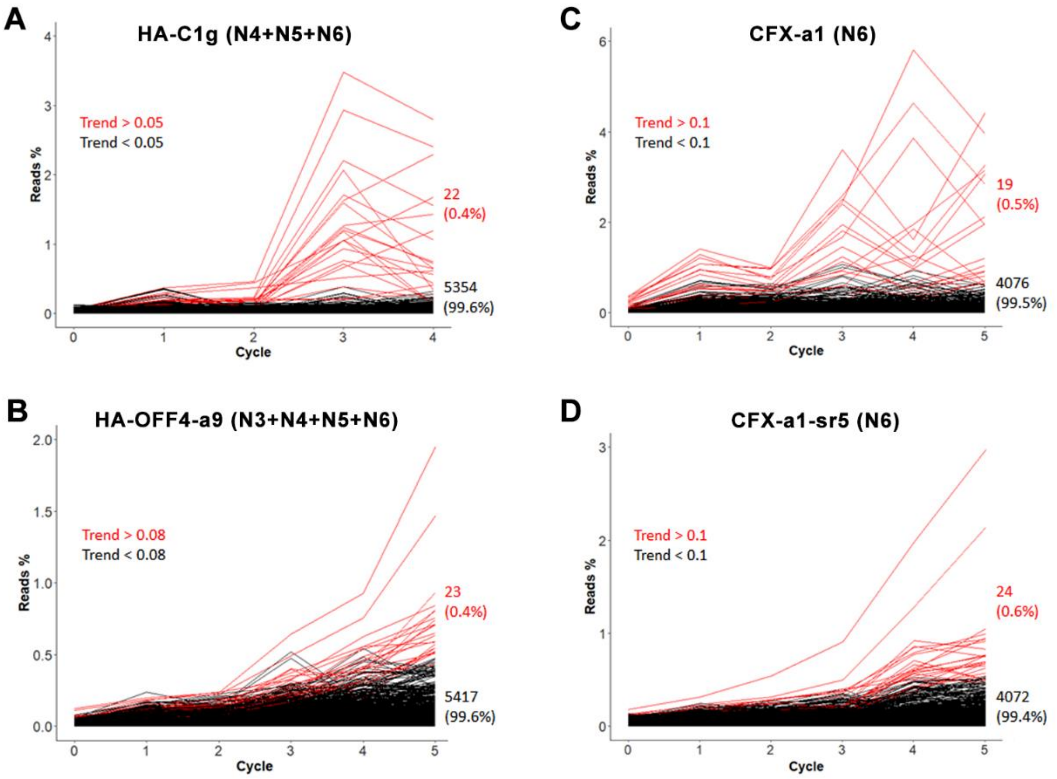

Supplementary Figure S7. Enrichment trend of individual variants through the sorting cycles. (**A**) Histamine ON-riboswitch library. (**B**) Histamine OFF-riboswitch library. (**C**) Ciprofloxacin ON-riboswitch library based on CFX-a1. (**D**) Ciprofloxacin ON-riboswitch library based on CFX-a1-sr5.

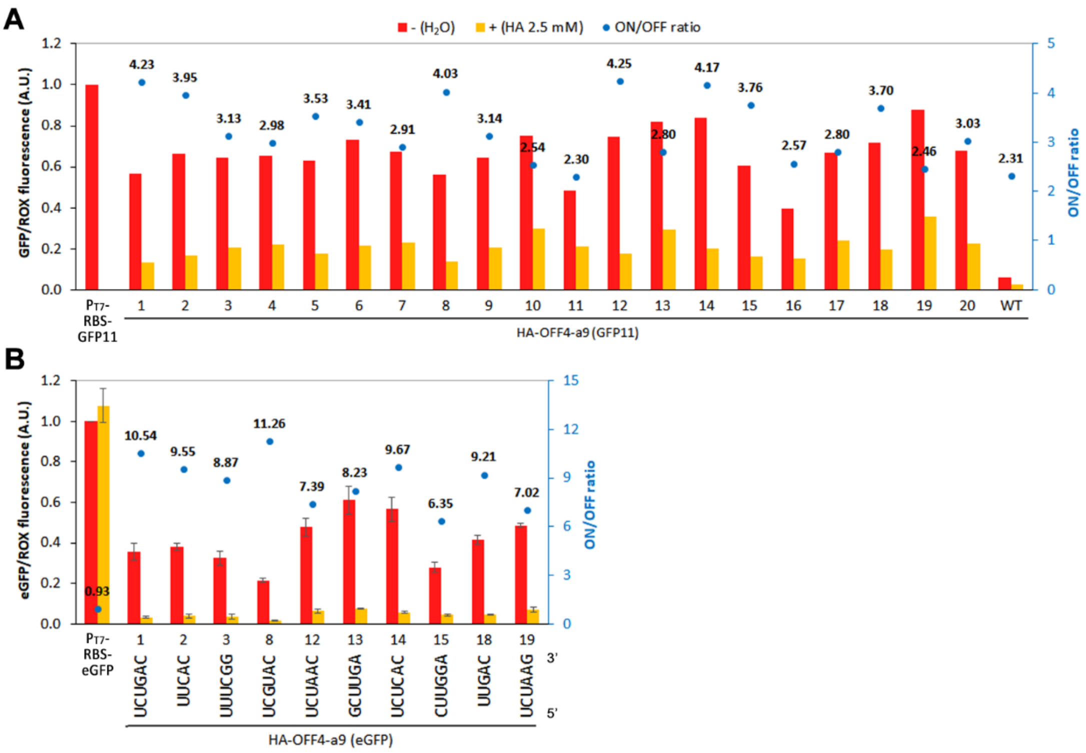

Supplementary Figure S8. Screening of histamine OFF-switches. The parental construct (HA-OFF4-a9) is denoted as WT. (**A**) Primary screening of the riboswitch variants using GFP11/GFP1–10 (split GFP) assay. Performed once for rapid screening. (**B**) Secondary screening of the riboswitch variants fused to full-length eGFP as a reporter gene. The assay was performed in three independent replicates for each variant with the error bars representing standard deviations. The expression levels were normalized by a no-riboswitch control P_{T7}-RBS-eGFP in the absence of histamine. The numbers of the variants correspond to their respective rankings based on the enrichment trend. HA: histamine.

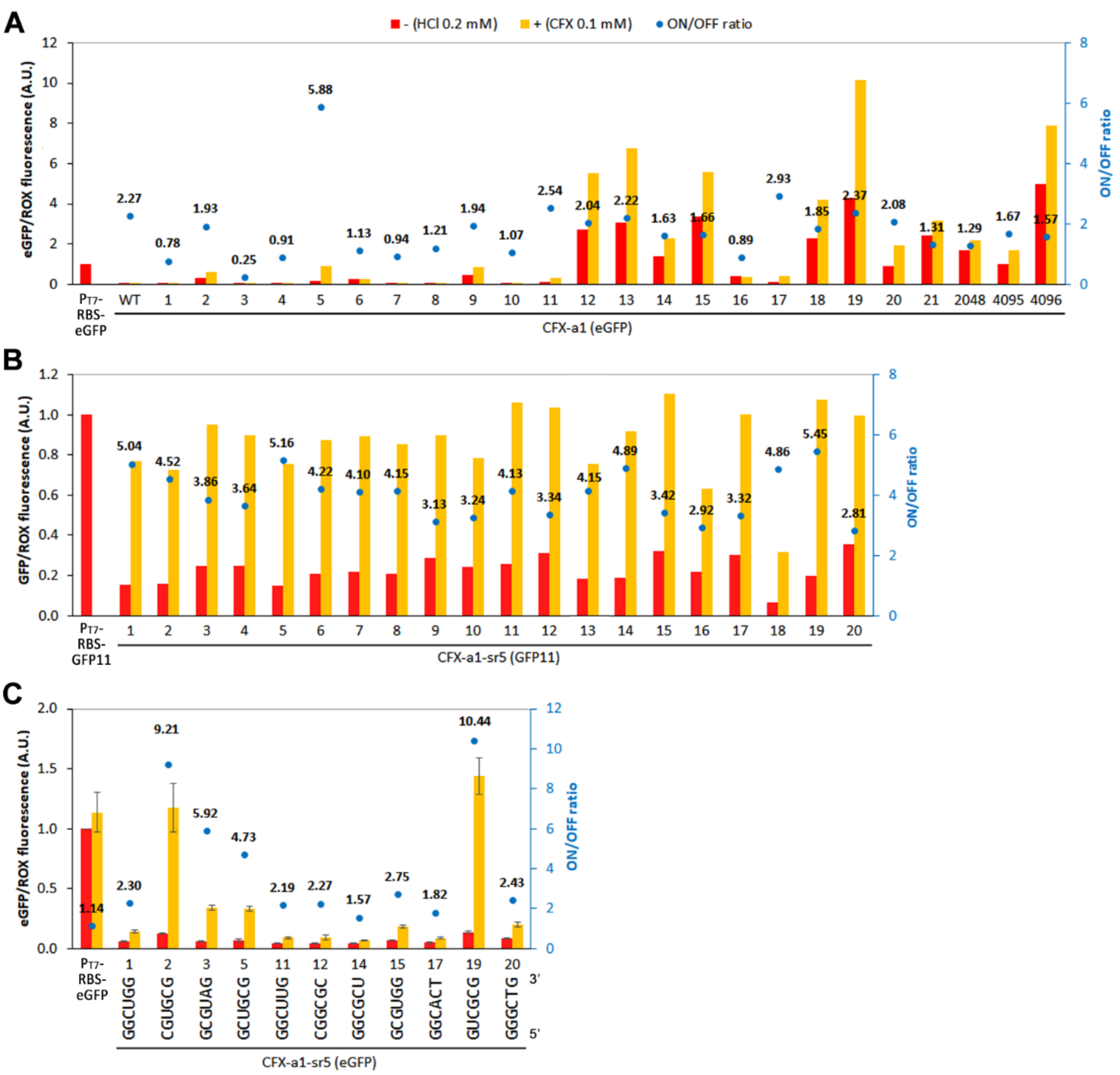

Supplementary Figure S9. Screening of ciprofloxacin ON-switches. The original parental construct (CFX-a1) is denoted as WT. The variant number 5 of the first screening (**A**) was renamed CFX-a1-sr5 and used as the new parental sequence for the next round of sorting cycles and screening (**B**, **C**). (**A**) Primary screening of the riboswitch variants (based on CFX-a1) using GFP11/GFP1–10 (split GFP) assay. Performed once for rapid screening. (**B**) Primary screening of the riboswitch variants (based on CFX-a1-sr5) using GFP11/GFP1–10 (split GFP) assay. Performed once for rapid screening. (**C**) Secondary screening of the riboswitch variants fused to full-length eGFP as a reporter gene. The assay was performed in three independent replicates for each variant with the error bars representing standard deviations. The expression levels were normalized by a no-riboswitch control P_{T7}-RBS-eGFP in the absence of ciprofloxacin. The numbers of the variants correspond to their respective rankings based on the enrichment trend. CFX: ciprofloxacin.

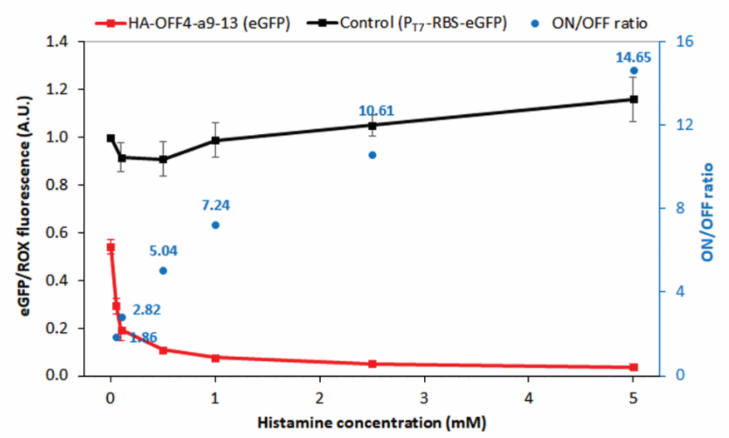

Supplementary Figure S10. Dose-dependence of HA-OFF4-a9-13 riboswitch (red) and a no-riboswitch control P_{T7}-RBS-eGFP (black). The ON/OFF ratios were corrected for the non-specific effects of histamine on P_{T7}-RBS-eGFP. The error bars represent the standard deviation of three independent assays.

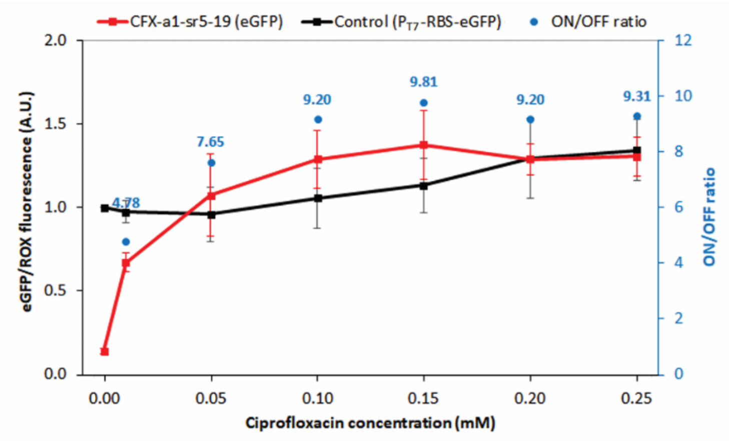

Supplementary Figure S11. Dose-dependence of CFX-a1-sr5-19 riboswitch (red) and a no-riboswitch control P_{T7}-RBS-eGFP (black). The ON/OFF ratios were corrected for the non-specific effects of ciprofloxacin on P_{T7}-RBS-eGFP. The error bars represent the standard deviation of three independent assays.

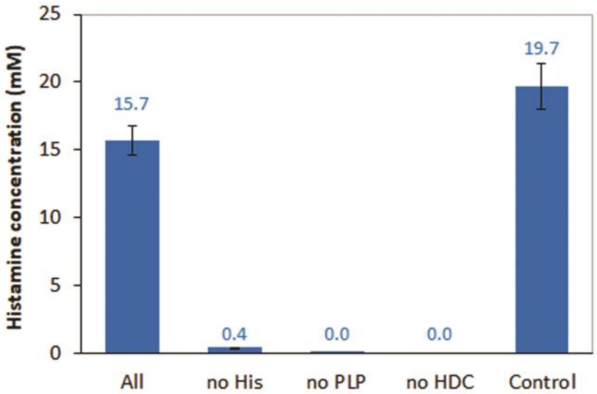

	All	no His	no PLP	no HDC	Control
HDC template (10 nM)	+	+	+	-	-
PLP (20 µM)	+	+	-	+	-
L-Histidine (20 mM)	+	-	+	+	-
Histamine (20 mM)	-	-	-	-	+

Supplementary Figure S12. Histamine decarboxylase (HDC) activity in a CFPS system. The enzyme and its cofactor, pyridoxal phosphate (PLP), are both essential for converting L-histidine (substrate) into histamine. The small amount of histamine produced in the sample without L-histidine (no His) comes from the L-histidine present as part of the amino acids pool of the CFPS reaction. The HDC enzyme was directly produced inside the PURE*frex* 1.0 reaction mix (5 µl total reaction volume) containing the DNA template P_{T7}-*hdc*-6xHis encoding the HDC (10 nM), PLP (20 µM), and L-histidine (20 mM). A histamine solution (20 mM) was used as a positive control. The samples were incubated at 37°C for 4 h, and then the histamine produced during the CFPS reaction was measured with a "Histamine Test" colorimetric kit (Kikkoman Biochemifa) and Infinite M1000 Pro (Tecan) plate reader (Abs_{470nm}). The error bars represent the standard deviation of three independent assays.

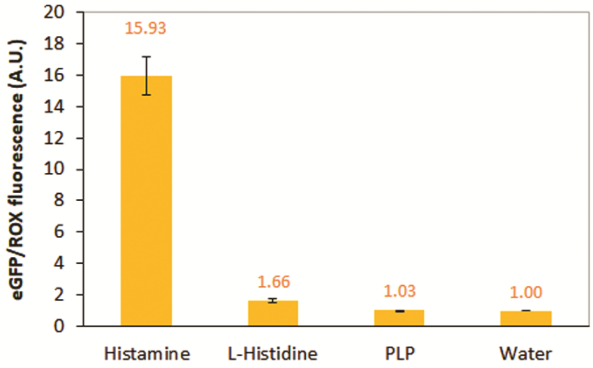

Supplementary Figure S13. HA-C1g-19 riboswitch is selectively activated by histamine. L-histidine due to its structural similarity to histamine slightly activates the riboswitch but barely above the background level (Water), as the K_d of the aptamer for histamine (~371 nM) is two orders of magnitude smaller than for L-histidine (~23 µM) (Dwidar et al. *J. Am. Chem. Soc.* 2019, **141**, 11103-11114). Pyridoxal phosphate (PLP) has no effect on the riboswitch activation. The CFPS reaction was performed as described in Materials and Methods with a DNA template (20 nM) encoding eGFP controlled by the HA-C1g-19 riboswitch, and either histamine (5 mM), L-histidine (5 mM), or PLP (20 µM) as the ligand. The expression levels were normalized by that of the untreated sample (Water). The error bars represent standard deviation of three independent assays.
